# Decoding nature: multi-target anti-inflammatory mechanisms of natural products in the TLR4/NF-κB pathway

**DOI:** 10.3389/fphar.2024.1467193

**Published:** 2025-01-14

**Authors:** Yue Zhao, Jiacai Wu, Xiaolan Liu, Xu Chen, Juan Wang

**Affiliations:** ^1^ College of Pharmacy, Guilin Medical University, Guilin, China; ^2^ Key Laboratory of Pharmacognosy, College of Pharmacy, Guilin Medical University, Guilin, China; ^3^ College of Intelligent Medicine and Biotechnology, Guilin Medical University, Guilin, China; ^4^ Guangxi Key Laboratory of Molecular Medicine in Liver Injury and Repair, Guilin Medical University, Guilin, China

**Keywords:** natural products, NF-κB pathway, TLR4 pathway, inflammation, molecular pharmacology

## Abstract

Natural products are valuable medicinal resources in the field of anti-inflammation due to their significant bioactivity and low antibiotic resistance. Research has demonstrated that many natural products exert notable anti-inflammatory effects by modulating the Toll-like receptor 4 (TLR4) and nuclear factor kappa B (NF-κB) signaling pathways. The research on related signal transduction mechanisms and pharmacological mechanisms is increasingly being discovered and validated. However, there is currently a lack of comprehensive reviews focusing on the pharmacological mechanisms of natural products targeting the TLR4/NF-κB pathway for anti-inflammatory effects. In light of these considerations, this review comprehensively synthesizes recent research findings concerning the TLR4/NF-κB signaling pathway, including the translocation of TLR4 activation to lysosomes within the cytoplasm, the assembly of protein complexes mediated by ubiquitin chains K63 and K48, and the deacetylation modification of p65. These discoveries are integrated into the classical TLR4/NF-κB pathway to systematically elucidate the latest mechanisms among various targets. Additionally, we summarize the pharmacological mechanisms by which natural products exert anti-inflammatory effects through the TLR4/NF-κB pathway. This aims to elucidate the multitarget advantages of natural products in the treatment of inflammation and their potential applications, thereby providing theoretical support for molecular pharmacology research on inflammation and the development of novel natural anti-inflammatory drugs.

## 1 Introduction

Inflammation represents a nonspecific immune response of the body to pathogenic microorganisms, injurious substances, or other stimulating factors, serving as a fundamental component of the body’s immune defense mechanisms (Kotas and Medzhitov, 2015). Though self-limiting inflammation is a physiological response necessary for pathogen clearance, persistent inflammation is detrimental to the organs experiencing inflammation and can trigger systemic reactions in other organs. In particular chronic inflammatory diseases are considered as one leading cause of mortality worldwide, with over 50% of deaths attributed to inflammation-related diseases (Furman et al., 2019). For instance, sustained inflammation accelerates the progression of atherosclerosis and endothelial dysfunction and then results in joint deformities and loss of function in rheumatoid arthritis (RA) (Aletaha and Smolen, 2018).

Acute and chronic inflammatory reactions often necessitate pharmacological interventions to halt further progression. However, the side effects associated with general chemical anti-inflammatory drugs and antibiotic resistance cannot be ignored, such as weight gain, elevated blood pressure, an increased risk of cataracts and glaucoma, and gastrointestinal disorders (Kavanaugh and Wells, 2014). Furthermore, the issue of antibiotic resistance stemming from certain antibiotic-based anti-inflammatory drugs is a critical concern, with the World Health Organization (WHO) designating bacterial resistance as a major public health crisis (Leung et al., 2011) Underscoring the urgent need to identify anti-inflammatory medications with low adverse reactions and reduced resistance (Wagenlehner and Dittmar, 2022).

Over the past 3 decades, Natural products have emerged as a significant source of novel therapeutics for treating diseases (Newman and Cragg, 2007). In comparison to chemically synthesized drugs, natural product-based medications offer distinct advantages in terms of structural novelty, biocompatibility, and functional diversity, attributes that have been evolutied through extensive natural selection during evolution. Statistics reveal that more than 50% of drugs approved by the United States Food and Drug Administration (FDA) for market authorization from 1939 to 2016 have originated from natural products (Rodrigues et al., 2016). Natural products exhibit superior characteristics of multi-target modulation, broad adaptability, and high safety, showcasing immense potential in the realm of anti-inflammatory properties (HUANG et al., 2018).

With the continuous advancement of life science technologies, research on the pathways of action mechanisms of natural products has become indispensable, given the diverse structures and complex mechanisms of action of plant-based medicines. The NF-κB protein, a transcription factor that regulates the expression of numerous immune-related genes, is present in almost all animal cells (Cai et al., 2022). The TLR4/NF-κB pathway it is involved in plays a crucial role in mediating inflammatory responses, immune reactions, antimicrobial defense, and immune homeostasis. Upon exposure to inflammatory stimuli, such as IL-1, TNF-α or lipopolysaccharide (LPS), the NF-κB signaling pathway is activated. This activation leads to an increase in the expression of key proteins including MyD88, NF-κB inhibitory protein (IκB), and p65, resulting in the translocation of NF-κB to the nucleus where it binds to DNA and promotes the release of pro-inflammatory factors (Doyle and O'Neill, 2006; Gray et al., 2016; O'Neill and Bowie, 2007).

As mentioned earlier, the TLR4/NF-κB pathway plays a significant role in regulating inflammatory signaling. With the rapid development of life science technology, it is necessary to timely summarize the latest research on TLR4/NF-κB, to quickly understand the research status and development trends in this field. Therefore, this review systematically explores the interactions between key targets in the upstream and downstream pathways of TLR4/NF-κB, while also supplementing and incorporating the latest research findings. Such as the membrane translocation after TLR4 activation,the assembly of protein complexes mediated by ubiquitin chains K63 and K48 and the deacetylation of p65 inhibits NF-κB activity. In addition, given the limitations of current chemical anti-inflammatory drugs in clinical applications, this review systematically summarizes the molecular pharmacological mechanisms by which natural products exert anti-inflammatory effects through the TLR4/NF-κB pathway. This will provide an important theoretical basis for the development of natural anti-inflammatory drugs, deepen the understanding of drug mechanisms, guide future research directions, and promote advances in pharmacology.

## 2 Method

This comprehensive review was performed by searching PubMed, using a time-based filter to capture all potential studies from 2000 to 15 June 2024, and selecting 58 unique, representative, and innovative natural product articles for review, covering as comprehensively as possible various drug doses, modeling methods, and pharmacological mechanisms. We used two types of search strategies, the first type was applied in “TLR4/NF - κ B signaling pathway in inflammation”, the search included both “All Field” (TLR4/NF-κB, signal transduction) and “title/abstract” (inflammation, immunity).

The second type was applied in “Anti-inflammatory mechanism of Natural products in the TLR4/NF/κ B pathway”, the search included both “All Field” (TLR4/NF-κB, Natural products) and title/abstract (herb, plant compounds, mechanism, signaling pathway, inflammation). Use the logical conjunction ‘AND’ between the search term ‘All Field’ and ‘Title/Abstract’. All Fields and Title/Abstract were reasonably matched according to the actual search results. In addition, a supplementary search was conducted on the reference list of the included studies.

The botanical names mentioned in this review were cross-checked using the International Plant Names Index (IPNI 2023; www.ipni. org) and The Plant List (TPL 2013; www.theplantlist. org) databases, and the plant names used were “Accepted” in TPL.

## 3 The application of natural products in inflammation

In the field of inflammatory diseases, especially chronic inflammatory diseases, natural products not only exhibit similar therapeutic effects as steroidal anti-inflammatory drugs, but also have advantages such as low side effects, low drug resistance, and diverse biological activities. Consequently, an increasing number of advanced and innovative mechanisms are being explored by researchers. For instance, some natural products have been developed as immunosuppressants, offering unique targeting capabilities to inhibit immune responses and prevent organ rejection. One example is resveratrol, which activates SIRT1 (a deacetylase) to suppress the transcriptional activity of NF-κB, thereby reducing the production of pro-inflammatory cytokines (Jhou et al., 2017; Mendes et al., 2017). In addition, the establishment of the InflamNat database, in conjunction with network pharmacology, enables researchers to rapidly screen natural products with potential anti-inflammatory activity and predict their mechanisms of action (Zhang et al., 2022; Guo et al., 2024). On this basis, the structural characteristics of natural products also provide new insights for drug design. By studying the structure-activity relationships of these compounds, scientists can identify key structural features to optimize their pharmacological activity (Itoh and Inoue, 2019; Tew et al., 2020). At the same time, innovations in biotechnology such as single-cell multi omics applications, mass spectrometry imaging techniques, and the development of COX-1 and COX-2 inhibitors mimicking nonsteroidal anti-inflammatory drugs have highlighted the potential of natural products in regulating immune responses and inflammation (Nielsen and McNulty, 2019; Hou et al., 2022; Zhu et al., 2022).

**FIGURE 1:**
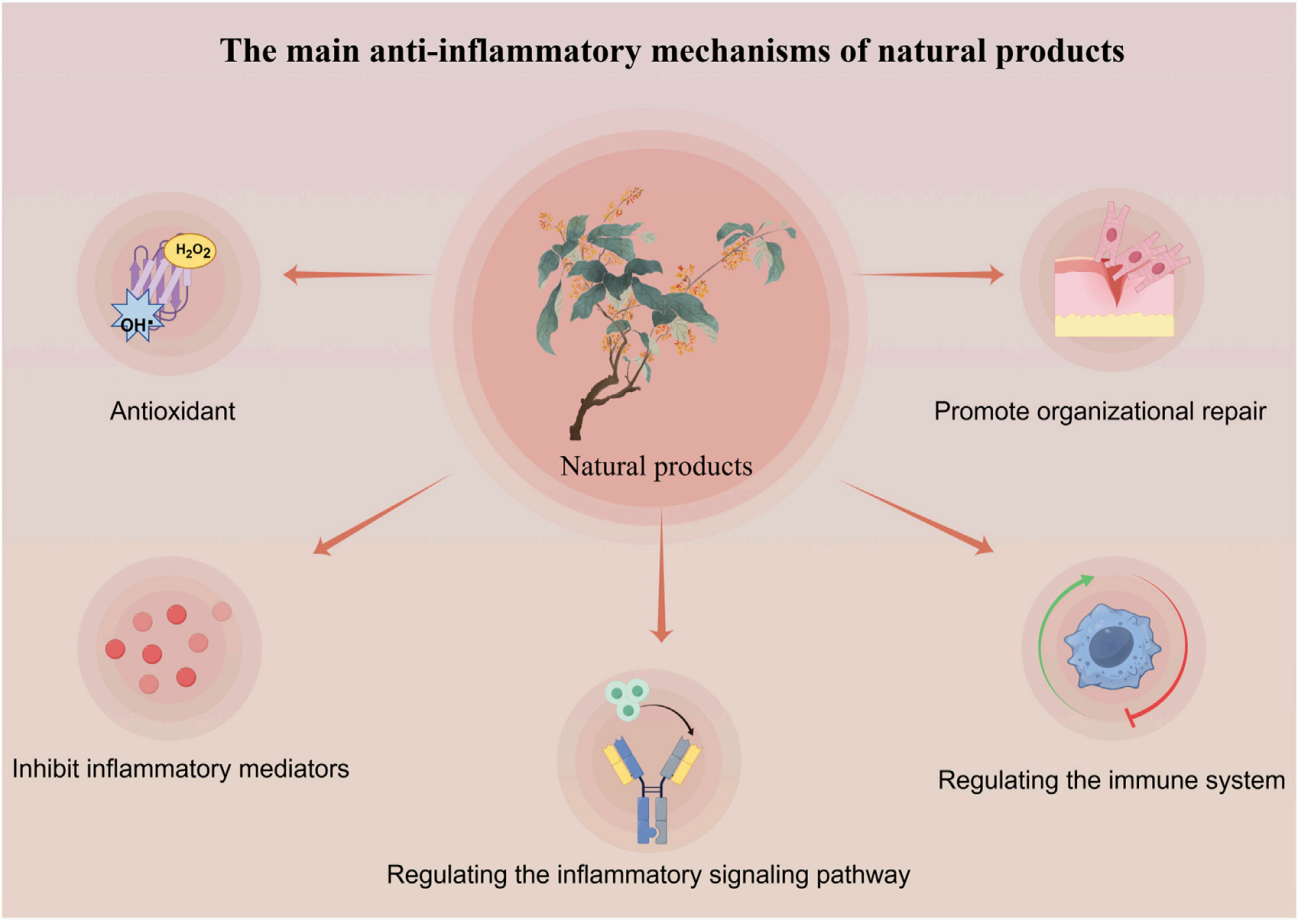
The main anti-inflammatory mechanisms of natural product.

Natural products exert anti-inflammatory effects through multi-target and multi-level effects. They can not only regulate common inflammatory signal transduction, but also work synergistically through antioxidant, tissue repair, and immune enhancement pathways (Zhang et al., 2010; Fang et al., 2024; Álvarez-Martínez et al., 2020) (As seen in Figure 1). For example, resveratrol activates SIRT1 to regulate the NF-κB signaling pathway, inhibiting the expression of pro-inflammatory cytokines. It also activates the Nrf2 signaling pathway, promoting the expression of antioxidant enzymes, enhancing cellular resistance to oxidative stress, and reducing oxidative damage associated with inflammation. Additionally, resveratrol promotes the expression of vascular endothelial growth factor (VEGF), facilitating angiogenesis and tissue repair (Baur and Sinclair, 2006). In contrast, conventional anti-inflammatory nonsteroidal drugs like aspirin primarily inhibit cyclooxygenase (COX) to exert their effects, while corticosteroids such as prednisone target a single protein, S6 kinase (S6K), to suppress the mTOR signaling pathway and diminish cellular responses to inflammatory stimuli (Mathiesen et al., 2014; Ng and Yeomans, 2018). Although these conventional therapies may offer more potent anti-inflammatory effects, their long-term use in chronic conditions such as rheumatoid arthritis and ulcerative colitis can lead to adverse reactions that are intolerable for patients. (Natural products in different anti-inflammatory mechanisms of TLR4/NF-κB pathway in Table 1).

**TABLE 1:** Natural products in different anti-inflammatory mechanisms of TLR4/NF-κB pathway.

Main mechanism	Botanical name Compound name	Ref.
Inhibiting TLR4 activated	*Scutellaria baicalensis* Georgi Baicalin	Fu et al. (2020)
	— Ferulic acid	Rehman et al. (2018)
Inhibition of TLR4 expression	*Lycium ruthenicum* Murray Polysaccharide	Peng et al. (2014)
	Sophora flavescens Aiton Oxymatrine	Lu et al. (2017a)
	*Glycyrrhiza uralensis* Fisch Glycyrrhetinic acid	Shi et al. (2020)
	*Glycine max (*L.) Merr Genistein	Jeong et al. (2014)
	— Quercetin	Zhao et al. (2021)
Inhibition of MyD88 Expression	*Forsythia suspensa* (Thunb.) Vahl Phillygenin	Hu et al. (2020)
	*Echinacea purpurea* (L.) Moenc*h* Echinacea polysaccharide	Zhang et al. (2020)
	*Anemarrhena asphodeloides* Bunge Timosaponin B-II	Zhang et al. (2015b)
Inhibition of TAK1 and IRAK1/4 Complex Activation	*Reynoutria japonica* Houtt Polydatin	Jiang et al. (2015)
	*Euphorbia pekinensis* Rupr Euphorbia Factor L2	Tang et al. (2021)
	*Panax ginseng* C. A. Mey Ginsenoside Rg5	Kim et al. (2012)
	*Cullen corylifolium* (L.) Medik Psoralen	Li et al. (2021)
	*Paris polyphylla* Sm Polyphyllin I	Wang et al. (2018a)
Inhibition of IKK Complex Activation	*Lycoris radiata* (L'Hér.) Herb Narciclasine	Shen et al. (2019)
	*Panax notoginseng* (Burkill) F.H.Chen Notoginsenoside R1	Jiao et al. (2021)
	— Genistein-3′-sodium sulfonate	Liu et al. (2021)
Inhibition of TRAF6 Expression and Ubiquitination	*Angelica gigas* Nakai Nodakenin	Rim et al. (2012)
	*Catharanthus roseus* (L.) G. Don Tabersonine	Zhang et al. (2018)
Inhibition of IκBα Degradation and Ubiquitination	*Myristica fragrans* Houtt Myrislignan	Jin et al. (2012)
	*Epimedium brevicornu* Maxim Icariside II	Zhou et al. (2019)
	*Waltheria indica* L Antidesmone	Lu et al. (2017b)
Deacetylation of p65	*Coptis chinensis* Franch Berberine	Zhang et al. (2023)
	— Resveratrol	Jhou et al. (2017)
Inhibition of p65 phosphorylation and nuclear translocation	*Rabdosia rubescens* (Hemsl.) H. Hara Oridonin	Li et al. (2018)
	*Panax notoginseng* (Burkill) F. H. Chen Ginsenoside B	Ran et al. (2018)
	*Siraitia grosvenorii* (Swingle) C. Jeffrey ex A. M. Lu and Zhi Y. Zhang Mogroside V	Han et al. (2024)
	*Lindera aggregate* (Sims) Kosterm Evodiamine	Fan et al. (2017)

**FIGURE 2:**
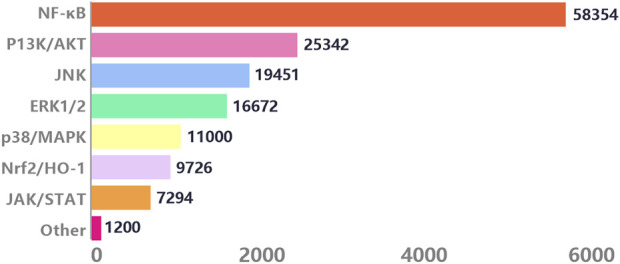
Journal analysis of inflammatory signaling pathways from 2019 to 2014.

With the in-depth research on the pathogenesis of inflammation by molecular biology, various signaling pathways triggering inflammation have attracted widespread attention. We conducted searches through the Web of Science, PubMed, and PubMed Central electronic databases, using terms ranging from subject headings (inflammation) to keywords (inflammation, signaling, mechanism), to retrieve research articles spanning 20 years from 2004 to 2024. Among the 15,000 articles screened, we found that among numerous anti-inflammatory signals, the NF-κB pathway appeared most frequently, accounting for 48.39%. This highlights the crucial role of the NF-κB signaling pathway plays in inflammatory signal transduction (Figure 2).

In conclusion, the TLR4/NF-κB pathway is essential in inflammation regulation. The screening and development of natural products with targeted anti-inflammatory effects represent a novel direction and strategy for treating inflammatory diseases, while also advancing the modernization and clinical application of natural products.

## 4 TLR4/NF-κB signaling pathway in inflammation

### 4.1 The structure and activation of TLR4

#### 4.1.1 TLR4 structure

TLR4, a member of the Toll-like receptor family, is a type I transmembrane protein discovered by Poltorak in 1998 (Fitzgerald and Kagan, 2020). Encoded by the TLR4 gene, it is expressed in various tissue cells, including monocytes and macrophages. TLR4 is mainly activated by LPS, a crucial immunoactivating factor derived from the surface of various bacteria (*Escherichia coli*, *Salmonella* spp.,*Pseudomonas aeruginosa*, *Vibrio cholerae*, *Enterobacter* spp. et al.). It is a characteristic component of the outer membrane of Gram-negative bacteria, playing a vital role in bacterial structure while also being recognized by the host immune system, thereby triggering a cascade of immune responses (Fitzgerald and Kagan, 2020).

The extracellular domain of TLR4 is a member of the leucine-rich repeat (LRR) family, playing a crucial role in ligand recognition and receptor dimerization. TLR4 exhibits a characteristic curved solenoid structure, with the myeloid differentiation protein 2 (MD2) intricately embedded within it (Kim et al., 2005; Kelley et al., 2013). When LPS binds to MD2, the LRR specifically recognizes the pattern recognition of related molecules (PAMP) and sends a signal to MD2, leading to TLR4 dimerization (Akashi et al., 2000). The intracellular domain of TLR4 is characterized by a conserved TIR domain that exhibits significant homology to the IL-1 receptor. Upon stimulation by extracellular MD2, the intracellular TIR domain is capable of initiating downstream NF-κB signaling transduction (Liu et al., 2014).

#### 4.1.2 Activation of TLR4 signaling pathway

**FIGURE 3:**
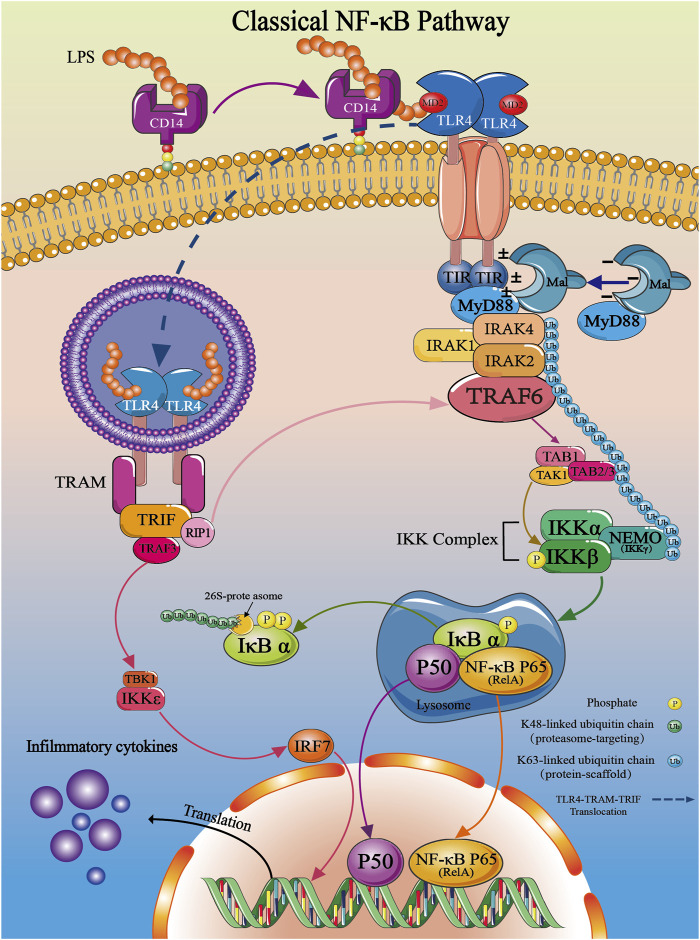
Overview of the TLR4/NF-κB classical signal pathway.

Upon invasion of the body by bacteria, the LPS present in the bacterial outer membrane is selectively identified and bound by TLR4, subsequently initiating the TLR4 signaling cascade. Lipid A, the hydrophobic component of LPS, initially attaches near the cell membrane as an endotoxin, and subsequently associates with a cluster of differentiation 14 (CD14) to form a complex. CD14, as the activation site of TLR4, can disassemble the LPS aggregates into monomeric molecules (Alarcón-Vila et al., 2020; Fitzgerald and Kagan, 2020; Alarcón-Vila et al., 2020). Subsequently, individual LPS molecules bind to MD-2, then cause the activation of TLR4 (Figure 3). After activation, the external structure and conformation of TLR4 is changed, leading to dimerization of the intracellular TIR domain. The dimerized TIR domain is recognized by TIR through TIR-TIR interactions. After recognition, it will lead to the occurrence of so-called “Myddosome” and “Triffosome” phenomena on the cell surface. The downstream signaling of the “Myddosome” involves the participation of MyD88 and MyD88 adapter-like (Haftcheshmeh et al.) proteins, along with the transitional role of TIR domain-containing adaptor protein (TIRAP) (Bonham et al., 2014; Akira, 2003). On the contrary, the “Triffosome” pathway is composed of TIR domain-containing adaptor-inducing interferon-β (TRIF) and TRIF-related adaptor molecule (TRAM) (Kagan et al., 2008). The different assembly complexes initiate different immune responses, thereby activating MyD88-dependent and the TRIF-dependent two TLR4-NF-κB pathways within the cells (Anderberg et al., 2017).

In the MyD88-dependent signaling pathway, the TIR domain carries a positive charge and binds to Mal protein with a negative charge through electrostatic forces. Upon binding, MyD88 is recruited to the activated TLR4 complex, initiating the MyD88-dependent signaling pathway. Activated interleukin receptor-associated kinases (IRAKs), such as IRAK1, IRAK2, and IRAK4, then activate tumor necrosis factor receptor-associated factor 6 (TRAF6), ultimately resulting in the activation of NF-κB (Figure 3) (Park and Lee, 2013; Tanimura et al., 2008). In another TRIF-dependent signaling pathway, TLR4 recruits TRIF and TRAM, which contains a TIR domain. As a substrate of protein kinase Cε (PKCε), TRAM allows it to target to the plasma membrane through its N-terminal myristoylation. Upon LPS stimulation, within 120 min, TRAM and TRIF translocate together with TLR4 into the endosome (Kobayashi et al., 2006; Rowe et al., 2006; Tanimura et al., 2008). During this stage, TRIF initiates the recruitment of tumor necrosis factor receptor-associated factor 3 (TRAF3) and receptor-interacting protein-1 (RIP1), resulting in TRAF3 self-ubiquitination and the formation of a complex with Tbk1 and IKKε. Subsequent phosphorylation of Tbk1 and IKKε leads to the activation of interferon regulatory factor 3 (IRF3) (Zanoni et al., 2011). IRF3 then forms homodimers and heterodimers with IRF7, binds to specific DNA sequences in the cell nucleus, and transcribes interferons (IFNs) and IFN-induced genes. Additionally, TRIF is capable of activating NF-κB by recruiting TRAF6 (late activation) (Figure 3) (Weiss and Barker, 2018). Through transient transfection experiments, Xiaoqin Su demonstrated the association between TRIF and TRAF6 (Sato et al., 2003; Jiang et al., 2004). Additionally, three TRAF6 binding motifs were found in TRIF, indicating the specific interaction between TRIF and TRAF6, resulting in the activation of NF-κB signaling pathways downstream (Ye et al., 2002). Hence, TIRAP and TRAM can be seen as ‘sorting adaptors’ that are involved in determining the subcellular placement of TLR signaling or the particular pathways that are triggered (Kagan, 2012; Di Lorenzo et al., 2022).

After activation of TRAF6, it exerts ubiquitination effects to activate IκB kinase (IKK) signaling: TRAF6, functioning as an E3 ubiquitin ligase, forms a ubiquitin ligase complex with the E2 conjugating enzyme complex UBC13-Uev1a, generating free K63-linked polyubiquitin chains (K63) to exert multiple ubiquitin effects (Deng et al., 2000; Wooff et al., 2004; Newton et al., 2008; Skaug et al., 2009; Xu et al., 2009). The formed K63 sequentially links IRAK1/2/4-TAK1-TAB1/2/3-NEMO (IKKγ) in the order of signal transduction, facilitating signal transduction among these proteins. Among them, transforming growth factor-β-activated kinase 1 (TAK1), upon receiving signals from free K63 polyubiquitin chains, activates TAK1-binding protein 1/2/3 (TAB1/2/3) signals to form a complex, leading to IKKβ phosphorylation, degradation of IKKβ, and initiation of NF-κB nuclear translocation (Figure 3) (Adhikari et al., 2007). Therefore, it is evident that unanchored free K63 chains play a pivotal role in the activation of IKK (Xia et al., 2009). Until now, four distinct types of ubiquitin chains-K11, K48, K63, and M1 chains have been implicated in the canonical NF-κB activation pathway (Figure 3) (Adhikari et al., 2007). Nevertheless, the precise mechanisms underlying the roles of K11 and M1 chains remain ambiguous, necessitating additional research in subsequent studies (Jin et al., 2008; Xu et al., 2009; Iwai, 2012).

### 4.2 The structure and activation of classic NF-κB

#### 4.2.1 The structure and inflammation of NF-κB

In mammals, five proteins, namely, p65 (RelA), RelB, c-Rel, p50 (NF-κB 1; and its precursor p105), and p52 (NF-κB 2; and its precursor p100) are classified as members of the NF-κB family. NF-κB protein family is a multifunctional transcription factor. Upon stimulation by LPS, oxidative stress, inflammatory cytokines, free radicals, or bacteria, the NF-κB protein family can specifically bind to κB sites in the promoter regions of various genes, promoting the transcription and translation of target genes, and it serves as an upstream regulatory protein for various downstream effector factors. Through the regulation of inflammatory cytokines, adhesion molecules, chemokines, and surface receptors, it participates in the regulation of immune responses, cell apoptosis, and tumorigenesis (Chen et al., 2011). NF-κB has classical and non-classical pathways two distinct signaling pathways, each pathway owns specific activation mechanisms (Sun, 2011; Cildir et al., 2016; Sun, 2017). The activation of the NF-κB pathway is initiated by diverse external stimuli that trigger the production and secretion of pro-inflammatory cytokines such as TNF-α, IL-1, IL-6, and so on (Zhang K. et al., 2015). In a state of homeostasis, the proteins p65 and p50 are confined to the cytoplasm by the IκB, resulting in the masking of their nuclear localization sequences and hindering their movement into the nucleus for DNA binding. The κB proteins possess a Rel-homology-domain (RHD) which facilitates their ability to bind to DNA, form dimers, and translocate into the nucleus. Among the five NF-κB family proteins, only p65, RelB, and c-Rel contain a transcriptional activation domain (TAD) that is responsible for activating target genes (Sehnert et al., 2020). The IKK complex consists of two homologous catalytic subunits, IKKα (IKK1) and IKKβ (IKK2), as well as a regulatory subunit, IKKγ (NF-κB essential modulator, NEMO) (Häcker and Karin, 2006). IKKβ plays a crucial role in initiating the classical NF-κB signaling pathway in response to pro-inflammatory cytokines and microbial stimuli, whereas IKKα predominantly governs the activation of the non-canonical NF-κB pathway (Israël, 2010; Ruland, 2011).

During homeostasis, the IκB confines the proteins p65 and p50 to the cytoplasm, thereby masking their nuclear localization sequences and preventing their entry into the nucleus to bind DNA. The κB proteins possess a Rel-homology-domain (RHD) which facilitates they binding to DNA, form dimers, and translocate into the nucleus. Within the NF-κB protein family, only p65, RelB, and c-Rel possess a transcriptional activation domain (TAD) that is accountable for triggering target genes. The IKK complex is made up of two similar catalytic components, IKKα (also named IKK1) and IKKβ (also referred to as IKK2), along with a regulatory subunit called IKKγ (also called NF-κB essential modulator or NEMO) (Häcker and Karin, 2006). IKKβ is essential for starting the traditional NF-κB signaling pathway when pro-inflammatory cytokines and microbial stimuli are present, while IKKα mainly controls the non-canonical NF-κB pathway activation.

#### 4.2.2 Activation of NF-κB signaling pathway

TAK1 phosphorylation leads to the binding of IKKs to the N-terminus of NEMO. Following this, IKKα/β/γ goes through oligomerization and phosphorylation, leading to the activation of IKKβ (Häcker and Karin, 2006; Ghosh and Hayden, 2008). When activated, IKKβ starts the process of phosphorylating IκB-α proteins, which then leads to their degradation through K48-linked ubiquitination by the 26S-proteasome (Gallo et al., 2017). The breakdown of IκB-α reveals the nuclear localization sequence (NLS) of cytoplasmic p65, which helps the p50/p65 dimer move into the nucleus (Hayden and Ghosh, 2008; Wertz and Dixit, 2010). In the nucleus, the NF-κB dimer (p50/p65) interacts with promoter regions of chromosomal loci, facilitating the transcription of target genes (Figure 3) (Hoffmann et al., 2006; Hayden and Ghosh, 2008).

## 5 Anti-inflammatory mechanism of natural products in the TLR4/NF/κ B pathway

The potential of Natural products in modulating the TLR4/NF-κB signaling pathway for the treatment of inflammation has garnered widespread attention among researchers. In inflammatory responses, Natural products effectively inhibit the expression of TLR4, thereby interrupting its recognition of pathogen-released LPS from bacteria, viruses, and other pathogens, thus preventing the activation of the NF-κB signaling pathway. Furthermore, Natural products can reduce the protein expression of pro-inflammatory factors, and inhibit IκB’s phosphorylation and ubiquitination processes, thereby influencing the signal transmission of the NF-κB pathway. Some Natural products even prevent NF-κB from entering the cell nucleus, hinder its binding with DNA, and consequently reduce the expression of pro-inflammatory factors, ultimately exerting anti-inflammatory effects (Figure 3). Based on the TLR4/NF-κB signaling pathway described earlier, we will categorize the mechanisms of action of Natural products at various targets and systematically summarize the molecular mechanisms by which Natural products exert anti-inflammatory effects in the TLR4/NF-κB pathway.

### 5.1 Inhibiting TLR4 activated by LPS

Fu et al. (2020) confirmed that baicalin extracted from Rhizoma Scutellaria baicalensis Georgi. [Lamiaceae] can reduce CD14 protein and mRNA expression through a CD14-dependent mechanism, inhibiting TLR4 activation and alleviating LPS-induced inflammatory responses. Furthermore, in a RAW264.7 cell model with CD14 knockout, the inhibition effect of baicalin on the inflammatory response induced by LPS was reversed. Additionally, Shafiq Ur Rehman et al. (2018) showed that ferulic acid (FA) can disrupt the binding site of the TLR4/MD2 complex, inhibiting TLR4 activation by LPS, which is crucial for triggering neuroinflammation through microglial cell activation.

### 5.2 Inhibition of TLR4 expression

Peng et al. (2014) found that the fruit of *Lycium ruthenicum* Murray. [Solanaceae] polysaccharide LRGP3 reduced the protein and mRNA expression of TLR4, leading to decreased levels of pro-inflammatory cytokines IL-1, IL-6, and TNF-α in the macrophages. Oxymatrine (OM), extracted from Root of *Sophora flavescens* Aiton. [Fabaceae], was shown to inhibit TLR4 levels in LPS-stimulated MS1 cells and the translocation of p65 to the cell nucleus, thereby reducing the release of IL-1β and alleviating the inflammatory response (Lu M. et al., 2017). Shi et al. (2020) found that glycyrrhetinic acid (GL) from the Rhizome of *Glycyrrhiza uralensis* Fisch. [Fabaceae] could inhibit TLR4 expression in the hepatitis virus (MHV) infection mouse, affect the HMGB1-TLR4 immune regulatory axis, and serve as a hepatoprotective factor in hepatic infectious diseases. Jeong et al. (2014) Jeong demonstrated that genistein attenuated the pro-inflammatory response of BV2 microglial cells stimulated by LPS, inhibited the binding of LPS to TLR4 in BV2 microglial cells and then weakened the downstream NF-κB signaling transduction. Le et al. (2020) found that quercetin exerted neuroprotective effects on HIBI mice by inhibiting TLR4 activation, thereby reducing oxidative stress and inflammatory responses in activated microglial cells.

### 5.3 Inhibition of MyD88 expression

Phillygenin (PhI) from Fruit of *Forsythia suspensa* (Thunb.) Vahl. [Oleaceae] is a lignan compound that can inhibit the expression of MyD88 protein, and then suppress LPS-induced pro-inflammatory responses and LX2 cell activation, thereby inhibiting liver fibrosis (Hu et al., 2020). Zhang et al. (2020) investigated that *Echinacea* polysaccharide (EP) from the Root of *Echinacea purpurea*  (L.) Moench. [Asteraceae] alleviated LPS-induced lung injury by inhibiting MyD88 expression and downregulating the TLR4/NF-κB signaling pathway. Zhang T. et al. (2015) discovered that timosaponin B-II (TB) from the Rhizome of *Anemarrhena asphodeloides* Bunge. [Asparagaceae], a major bioactive component in *anemarrhena asphodeloides*, inhibited MyD88 expression in LPS-induced ALI mice.

### 5.4 Inhibition of TAK1 and IRAK1/4 complex activation

Jiang et al. (2015) found polydatin (PD) from the Root of *Polygonum Reynoutria japonica* Houtt. [Polygonaceae] inhibit the activation of IRAK1 and IRAK4, regulate NF-κB signal transduction, and alleviate acute lung injury in mice. Tang et al. (2021) demonstrated that *Euphorbia* factor L2 (EFL2) extracted from the Seed of *Euphorbia pekinensis* Rupr. [Euphorbiaceae] inhibited IRAK4 activation and IKK phosphorylation, significantly downregulating the IRAK4-IKKβ-IRF5 and NF-κB signaling pathways to treat rheumatoid arthritis. Kim et al. (2012) found that ginsenoside Rg5 from the Root of Panax ginseng C.A.Mey. [Araliaceae], inhibited IRAK-1 phosphorylation induced by LPS, and promoted the degradation of IRAK1 and IRAK4, thereby blocking NF-κB signal transduction and improving lung inflammation. Psoralen, from the Fruit of *Cullen corylifolium* (L.) Medik. Psoralen [Leguminosae], was found to downregulate IRAK4 protein expression in an inflammation model of human periodontal ligament cells induced by Porphyromonas gingivalis LPS (P. gingivalis LPS), and then downregulate proteins in the TLR4 and NF-κB signaling pathways to treatment and prevention of periodontitis (Li et al., 2021). Polyphyllin I (PPI), a major component of the classic anti-inflammatory herb Rhizome *Paris polyphylla* Sm. [Melanthiaceae], was studied by Wang Q. et al. (2018). They found that when PPI inhibited the overexpression of IRAK1, TRAF6 and Tak1, it suppressed NF-κB transcription, inhibiting the production of pro-inflammatory mediators mediated by NF-κB in activated macrophages and improving synovial inflammation in CIA mice.

### 5.5 Inhibition of IKK complex activation

Shen et al. (2019) isolated narciclasine (NCS) from the aerial and bulb of Lycoris radiata (L'Hér.) Herb. [Amaryllidaceae], which can inhibit IKK/β phosphorylation in LPS-induced macrophages, thereby preventing the activation of the IKK complex. Similarly, *notoginsenoside* R1 (NG-R1) from the rhizome and root of *Panax notoginseng* (Burkill) F.H.Chen. [Araliaceae] inhibits the inflammatory cytokine production by improving the phosphorylation of IKKα/β and P65, as well as the nuclear translocation of P65, thus exerting anti-rheumatoid arthritis effect in TNF-Tg mice (Jiao et al., 2021). Liu et al. (2021) revealed that genistein-3′-sodium sulfonate (GSS) reduces IKK expression, and inhibits IκB degradation and P65 phosphorylation, which demonstrates that a neuroprotective effect of GSS on ischemic stroke rats.

### 5.6 Inhibition of TRAF6 expression and ubiquitination

Nodakenin is a coumarin isolated from the root of *Angelica gigas* Nakai. [Apiaceae], was discovered to inhibit the ubiquitination of TRAF6. This caused an obvious reduction in the binding of p-TAK1 and TRAF6 induced by LPS, thereby inhibiting the degradation of IκBα and the transcriptional activity of NF-κB (Rim et al., 2012). Eupatolide, a novel active component from the dry head inflorescence of *Inula britannica* L. [Asteraceae]*,* can induce the proteasomal degradation of TRAF6. By inducing the polyubiquitination of TRAF6 through Lys48 linkage, thereby suppressing the release of inflammatory factors and mitigating the inflammatory process (Lee J. et al., 2010). Likewise, Tabersonine (TAB), an alkaloid from the herb of *Catharanthus roseus* (L.) G. Don [Apocynaceae], has shown inhibitory effects on TRAF6 ubiquitination. Depeng et al. (Zhang et al., 2018) initially observed through luciferase assays that TAB treatment significantly inhibits NF-κB luciferase activity driven by TRAF6. Subsequently, immunoprecipitation experiments confirmed that TAB can reduce the K63-linked polyubiquitination of TRAF6.

### 5.7 Inhibition of IκBα degradation and ubiquitination

Myrislignan, isolated from the seed of *Myristica fragrans* Houtt. [Myristicaceae], was reported can inhibit the ubiquitination degradation of IκBα and nuclear translocation (Jin et al., 2012). Zhou et al. (2019) found that icariside II (ICS II) from the leaf of *Epimedium brevicornu* Maxim. [Berberidaceae] inhibits IκB degradation, and modulates the TLR4/MyD88/NF-κB pathway to attenuate endotoxin-induced neuroinflammation. Lu X. et al. (2017) found that treatment with Antidesmone from the root and stem of *Waltheria indica* L. [Malvaceae] could inhibit the degradation of IκBα in lung tissue nuclear extracts, reduce p65 protein levels, can inhibit inflammation on stimulated macrophages and thereby prevent acute lung injury by regulating NF-κB signaling pathways.

### 5.8 Deacetylation of p65

Natural products, through deacetylation of NF-κB p65, inhibition of p65 transcriptional activity and have anti-inflammatory effects. *Coptis chinensis* Franch. [Ranunculaceae] is a classic anti-inflammatory traditional Chinese medicine, and berberine isolated from it has been found to have significant anti-inflammatory activity in recent years (Zhang et al., 2017; Li et al., 2020; Tew et al., 2020; Haftcheshmeh et al., 2022). Shuchen Zhang et al. show that berberine reduces the acetylation of NF-κB subunit p65 at site Lys310 (p65 Lys310), leading to the inhibition of NF-κB translocation and transcriptional activity to suppress the expressions of inflammatory factors (Zhang et al., 2023). Jyun Pei Jhou et al. found that resveratrol-mediated transcriptional enhancement of the Fcγ RIIB gene resulted in reduced binding of acetylated p65 NF-κB (K310) and P-p65 NF - κ B (S468) to the −480 promoter region of Fcgr2b gene, improving lupus erythematosus (Jhou et al., 2017).

### 5.9 Inhibition of p65 phosphorylation and nuclear translocation

Oridonin (Ori), a diterpenoid compound isolated from the dry aboveground parts of Rabdosia rubescens (Hemsl.) H. Hara. [Lamiaceae], exhibits diverse biological activities (Dong et al., 2014; Wang et al., 2016; Liu et al., 2016), besides the inhibition of the phosphorylation of IκBα and p65, it also suppressing NF-κB DNA binding activity (Li et al., 2018). Zhao et al. (2016) demonstrated that oridonin dose-dependently inhibits p65 nuclear translocation, alleviating sepsis-induced renal injury. Similarly, Ran et al. (2018) showed through immunofluorescence analysis that pretreatment with ginsenoside B significantly blocks p65 nuclear translocation in rat chondrocytes. Han et al. (2024) demonstrated that after treatment with mogroside V from the fruit of *Siraitia grosvenorii* (Swingle) C. Jeffrey ex A.M.Lu & Zhi Y. Zhang. [Cucurbitaceae], the levels of P-p65 in mouse lung tissues were reduced, indicating the therapeutic efficacy of MV in alleviating lung inflammation induced by asthma. Evodiamine (EVO), is a natural alkaloid from the root tuber of *Lindera aggregate* (Sims) Kosterm. [Lauraceae], improves abnormal states of lung and intestinal tissues by inhibiting NF-κB expression *in vivo*, significantly reducing mortality induced by yeast polysaccharides. The mechanism may involve the inhibition of IκBα phosphorylation and p65 nuclear translocation, attenuating yeast polysaccharide-induced p65 DNA binding activity (Fan et al., 2017).

There are many other Natural products with anti-inflammatory effects. The anti-inflammatory pharmacological mechanisms of various Natural products are summarized in Table 2.

**TABLE 2:** Anti-inflammatory pharmacology of Natural products in TLR4/NFκB pathway.

Family	Botanical name/Compound name	Application	Doses/Duration	*In vitro*/*In vivo* models	Mechanisms	Ref.
Solanaceae	*Lycium ruthenicum Murray* polysaccharide	*In vitro*	10–80 μg/mL for 24 h	LPS-induced RAW264.7 inflammatory model	Reduces TLR4 protein expression; Inhibits IκBα degradation	Peng et al. (2014)
Solanaceae	*Lycium barbarum* L polysaccharides	*In vivo*	400, 800, 1,600 mg/kg, i.g., daily, for 8 weeks	The Wistar rats mode of liver fibrosis was established by gavage olive oil with 40 v/v% CCl4	Reduces TLR4 protein expression; Inhibits p65 phosphorylation	Gan et al. (2018)
Polyporaceae	*Ganoderma lucidum* (Leyss. ex Fr.) Polysaccharide peptide	*In vivo*	100, 200 mg/kg, i.g., daily, for 35 days	The Wistar rats were intradermally injected with bovine type II collagen in the tail base to establish the collagen-induced arthritis (CIA) model	Reduces p65 protein expression; Inhibits IκBα, p65 phosphorylation; Inhibits IκBα degradation	Meng et al. (2023)
Apiaceae	*Angelica sinensis (Oliv.) Diels* Aboveground part polysaccharide	*In vivo* *In vitro*	50, 100, 150 mg/kg, i.p., daily, for 14 days 5, 10, 20 μg/mL for 12 h	The C57BL/N mice model of colonic inflammation was established by intraperitoneal injection of DSS. LPS-induced IPEC-J2 cells inflammatory model	Reduces TLR4 protein expression; Inhibits p65 Phosphorylation	Zou et al. (2023)
Asteraceae	Echinacea purpurea (L.)Moench Echinacea polysaccharide	*In* *vivo* *In vitro*	5, 10 mg/kg, i.p., for 24 h 100 μg/mL for 1 h	The C57BL/6 mice mode of acute lung injury was established by intraperitoneal injection of LPS. LPS-induced RAW264.7 inflammatory model	Reduces TLR4, MyD88, p65 protein expression; Increases IκBα protein expression; Inhibits IκBα and p65 phosphorylation	Zhang et al. (2020)
Lamiaceae	Rabdosia rubescens (Hemsl.) H. Hara Oridonin	*In vivo* *In vitro*	10 mg/kg, i.p., daily, for 12 weeks 2.5, 5, 10, 20 μM for 12, 24, 48 h	The SD rats model of insulin resistance was established by fed a high-fat diet; The insulin resistance model rats were converted into DM rats by a single intraperitoneal injection of STZ. A rat mesangial cell line (HBZY-1)	Reduces TLR4 protein expression; Inhibits IκB-α, p65 phosphorylation; Prevents p65 nuclear translocation	Li et al. (2018)
Betulaceae	*Betula platyphylla Sukaczev* Betulin	*In vivo* *In vitro*	4, 8 mg/kg, i.p., for 24 h 2, 4, 8 μg/mL for 1 h	Sepsis-induced acute lung injury in SD rats was established by CLP. LPS-induced rat HBZY-1 mesangial cell line inflammatory model	Reduces TLR4 mRNA expression; Inhibits IκB-α, IKK-α/β and p65 phosphorylation; Prevents p65 nuclear translocation	Zhao et al. (2016)
Berberidaceae	Epimedium brevicornu Maxim. Icariside II	*In vivo*	3, 10 mg/kg, i.g., for 14 days	The SD rats model of acute neuroinflammation was established by intraventricular injection of LPS.	Reduces TLR4、MyD88 and TRAF6 protein expression; Inhibits IκB degradation	Zhou et al. (2019)
Paeoniaceae	Paeonia suffruticosa var. papaveracea (Andrews) A. Kern. Paeoniflorin-6′-O-benzene sulfonate (CP-25)	*In vivo*	35 mg/kg, i.g., daily, for 40 days	The DBA/1 mice model of CIA was established by intradermal injection of the chicken CII mixture was then emulsified with Freund’s complete adjuvant into the back and base of the tail	Reduces TRAF2 protein expression; Inhibits p65 phosphorylation	Shu et al. (2019)
Melanthiaceae	Paris polyphylla Sm. Polyphyllin I	*In vivo* *In vitro*	1 mg/kg, i.g., daily, for 7 weeks; 0.25, 0.5, 1 μM for 3 h	The CIA model of C57BL/6 and DBA/1J mice LPS and IFN- γ Stimulate primary bone marrow-derived macrophages (BMMs) and peritoneal macrophages (PEMs)	Reduces p65, MyD88, IRAK1, TRAF6 and TAK1 protein expression; Inhibits IKK-α/β and p65 phosphorylation; Prevents p65 nuclear translocation	Wang et al. (2018a)
Polygonaceae	*Reynoutria japonica* Houtt Polydatin	*In vivo* *In vitro*	20, 80 mg/kg, i.p., for 1 h 2, 4, 8 μM for 2 h	The Balb/c mice mode of acute lung injury was established by intraperitoneal injection of LPS. LPS-induced human bronchial epithelial BEAS-2B cells	Reduces TLR4, MyD88 and IRAK-1 protein expression; Inhibits IKKα/β, IκB-α and p65 phosphorylation	Jiang et al. (2015)
Euphorbiaceae	*Euphorbia pekinensis* Rupr. Euphorbia Factor L2	*In vivo* *In vitro*	15, 40 mg/kg, i.p., for 8 days 0.1–100 μM for 20 h	The K/BxN mice model of K/BxN serum metastatic arthritis (STA) was established by intraperitoneal injection of K/BxN mouse serum RAW264.7 cells Bone marrow cells were harvested from the tibias and femurs of C57BL/6 mice	Reduces p65, IKK-α/β, IκB-α, IRAK4, and IKK-β protein expression; Inhibits IKK-α/β, IκB-α, IKKβ and p65 phosphorylation; Prevents p65 nuclear translocation	Tang et al. (2021)
Zingiberaceae	*Curcuma long*a L Curcumin salicylate monoester	*In vivo*	0.1, 0.2 mmol/kg, i.p., daily, for 14 days	The SD rats with Freund’s complete adjuvant (FCA)-induced arthritis (Ye et al.).	Inhibits IκB-α, IKKs and p65 phosphorylation	Zhang et al. (2019)
Lamiaceae	Scutellaria baicalensis Georgi Baicalin	*In vivo* *In vitro*	100 mg/kg, i.p., for 3 days 6.25–200 μM for 24 h	The male Balb/c and C57BL/6 mice model of ulcerative colitis (UC) was established by intraperitoneal injection of DSS or LPS. LPS-induced RAW264.7 inflammatory model	Reduces CD14, MyD88 protein expression; Inhibits p65 phosphorylation	Fu et al. (2020)
—	— Quercetin	*In vivo*	30, 60 mg/kg, i.g., daily, for 6 Weeks	The SD rats of diabetes were established by intravenous streptozotocin	Reduces TLR4, MyD88 and p65 protein expression	Zhao et al. (2021)
—	— Quercetin	*In vivo* *In vitro*	50 mg/kg, i.p., for 0, 24, 48 h 0–50 μM for12 h	Establishment of neonatal hypoxic-ischemic brain injury (HIBI) mice model The mouse BV2 microglial cells were incubated in a hypoxic chamber containing 1% O_2_/5% CO_2_/94% N_2_ for 3 h	Reduces TLR4, MyD88 and p65 protein expression	Le et al. (2020)
Leguminosae	*Cullen corylifolium* (L.) Medik Psoralen	*In vitro*	3.125, 6.25, 12.5, 25 μg/mL for 24 h	Primary hPDLCs were obtained and cultured from the ligament tissues in the middle of the premolar roots using a tissue explant method	Reduces TLR4, IRAK4 and P-p65 protein expression	Li et al. (2021)
Fabaceae	Glycine max (L.) Merr. Genistein	*In vitro*	25, 50 μM for 24 h	LPS-induced BV2 microglia inflammatory model	Reduces TLR4, MyD88 and p65 protein expression; Inhibits IκB-α degradation	Jeong et al. (2014)
—	— Genistein-3′-sodium sulfonate	*In* *vivo* *In vitro*	1 mg/kg, sublingual vein injection for 110min 10 μM for 24 h	The SD rats of transient middle cerebral artery occlusion and reperfusion (tMCAO) were established LPS-induced BV2 microglial cells as *in vitro* model	Reduces IKK and p65 protein expression; Inhibits IκB-α ubiquitination; InhibitsIKK and p65 phosphorylation	Liu et al. (2021)
—	— Hesperetin	*In vivo* *In vitro*	50 mg/kg, i.g., daily, for 5 Weeks 50 μM for 24 h	The C57BL/6 N mice mode of oxidative brain damage was established by intraperitoneal injection of LPS. LPS-induced mouse hippocampal (HT-22) and murine microglia (BV2) cell (*In vitro* model)	Reduces TLR4 protein expression; Inhibits p65 phosphorylation	Muhammad et al. (2019)
Rutaceae	Citrus × aurantium L. Naringenin	*In vivo*	10–50 mg/kg, p.o., daily, for 21 days	The Wistar rats mode of cerebral ischemia was established by MCAO.	Reduces p65 protein expression	Raza et al. (2013)
Piperaceae	*Piper nigrum* L Alkaloids	*In vivo* *In vitro*	50 mg/kg, i.g., for 5 h 0–8 μM for 24 h	The ICR mice mode of acute paw edema was established by injected into the right hind paw of carrageenan suspension (100 μL per mouse) LPS-induced RAW264.7 inflammatory model	Reduces IKKα/β protein expression; Inhibits IKKα/β, IκB-α and p65 phosphorylation; Inhibits IκB-α degradation	Pei et al. (2020)
Rubiaceae	Nauclea officinalis Pierre ex Pit. Strictosamide	*In vitro*	0–200 μM for 24 h	LPS-induced RAW264.7 inflammatory model	Inhibits IKKα, IκB-α, p65 phosphorylation	Li et al. (2017)
Lauraceae	*Lindera aggregate* (Sims) Kosterm Evodiamine	*In vivo* *In vitro*	10, 15 mg/kg, i.p., for 30 h 25, 50, 100 μM for 7 h	The C57BL/6J mice model of non-septic shock was established by intraperitoneal injection of zymosan Collect peritoneal macrophages from C57BL/6J mouse injected intraperitoneally with mercapto acetate broth	Inhibits IκB-α, p65 phosphorylation; Inhibits IκB-α degradation; Prevents p65 nuclear translocation	Fan et al. (2017)
Apocynaceae	Catharanthus roseus (L.) G.Don Tabersonine	*In vivo* *In vitro*	10–40 mg/kg, i.p., for 6 h 1–10 μM for 24 h	The C57BL/6 mice mode of acute lung injury was established by tracheal instillation of LPS Collect peritoneal macrophages induced by thioglycollate broth in the abdominal cavity of C57BL/6J mice	Inhibits TRAF6 ubiquitination; Prevents p65 nuclear translocation	Zhang et al. (2018)
Nitrariaceae	Peganum harmala L. Harmine	*In vivo* *In vitro*	30 mg/kg, i.p., for 1 day 2–50 μM for 24 h	The ICR mice model of inflammation by intraperitoneal injection of LPS. LPS-induced RAW264.7 inflammatory model	Prevents p65 nuclear translocation	Liu et al. (2017)
Malvaceae	*Waltheria indica L* Antidesmone	*In vivo* *In vitro*	2–8 mg/kg, i.p., for 12 h 0–200 μg/mL for 24 h	The Balb/c mice mode of acute lung injury was established by tracheal instillation of LPS. LPS-induced RAW264.7 inflammatory model	Inhibits IκB-α degradation; Prevents p65 nuclear translocation	Lu et al. (2017b)
Amaryllidaceae	*Lycoris radiata* (L'Hér.) Herb narciclasine	*In vitro*	0.001–0.016 µM for 24 h	LPS-induced RAW264.7 inflammatory model	Inhibits IκB-α degradation; Inhibits IκB-α, IKKα/β, p65 phosphorylation; Prevents p65 nuclear translocation	Shen et al. (2019)
Papaveraceae	Chelidonium majus L. Chelidonine	*In vivo* *In vitro*	1–9 mg/kg, i.g., daily, for 12 days 0–100 μM for 24 h	The Balb/c mice mode of inflammation was established by intraperitoneal injection of LPS. LPS-induced RAW264.7 inflammatory model	Reduces TLR4 protein expression; Inhibits of p65 nuclear translocation; Blocking IκB-α Phosphorylation and degradation	Liao et al. (2018)
Zingiberaceae	Curcuma longa L. Curcumin	*In vivo*	1–9 mg/kg, intestinal perfusion for 2 h	Lung lesion-induced Wistar rats mode was established by intestinal ischemia-reperfusion injury	Reduces TLR4 and MyD88 protein expression	Fan et al. (2015)
Zingiberaceae	Curcuma longa L. Curcumin	*In vivo*	5–20 μM for 24 h	The Wistar rats model of cerebral I/R injury was established by middle cerebral artery occlusion (MCAO, 1-h occlusion, and 24-h reperfusion)	Inhibits of p65 protein expression	Jin et al. (2007)
Ranunculaceae	*Aconitum carmichaelii* Debeaux Fuzi lipid-soluble alkaloids	*In vitro*	0–500 ng/mL for 24 h	IL-1β-induced human fibroblast-like synoviocytes-rheumatoid arthritis	Increases IκBα protein expression; Inhibits IκBα Phosphorylation; Inhibits of p65 nuclear translocation	Guo et al. (2022)
Fabaceae	Sophora flavescens Aiton Oxymatrine	*In vitro*	0–5 mg/mL for 24 h	Pancreatic microvascular endothelial cells and LPS induced inflammation to establish the cell model of microcirculation disturbances of acute pancreatitis	Reduces TLR4、MyD88 and p65 mRNA expression; Inhibits of p65 nuclear translocation	Lu et al. (2017a)
Fabaceae	Sophora flavescens Aiton Matrine	*In vivo* *In vitro*	100 mg/kg, i.g., daily, for 6 weeks 25 μM for 24 h	The SD rat model of RA was established by injected intra-dermally with 30 µg bovine type II collagen Using either mice splenic T cells stimulated with PMA/ionomycin or rat splenic T cells	Reduces p65and IκBα protein expression; Inhibits IκBα phosphorylation	Niu et al. (2017)
Convolvulaceae	Erycibe obtusifolia Benth. Scopoletin	*In vivo* *In vitro*	50–200 mg/kg, i.p., for 6 h 30–300 μM for 20 h	The acute inflammatory model resembling gout in ICR mice was induced by injecting MSU crystals into the pouch cavity MSU-induced RAW264.7 inflammatory model	Inhibits IκB-α,IKKα, p65 phosphorylation; Inhibits IκB-α degradation	Yao et al. (2012)
Apiaceae	Angelica gigas Nakai Nodakenin	*In vivo* *In vitro*	10, 20 mg/kg, i.p., for 1 h 25–100 μM for 24 h	The C57BL/6 mouse model of sepsis was established by intraperitoneal injection of LPS. LPS-induced RAW264.7 inflammatory mode	Reduces IRAK1 protein expression; Inhibits IκB-α Protein degradation; Inhibits TAK1、IKKα/β and IκB-α/β phosphorylation; Inhibits TRAF6 ubiquitination	Rim et al. (2012)
Schisandraceae	Schisandra chinensis (Turcz.) Baill. Schisandrin B	*In vivo* *In vitro*	50 μM, intra-articular injection., for 4 weeks 25–150 μM for 24 or 48 h	The SD rats were used to develop osteoarthritis by surgical resection of medial meniscus in knee joints Collecting chondrocytes from knee and hip cartilage collected from SD rats	Prevents p65 nuclear translocation	Ran et al. (2018)
—	— Ferulic acid	*In vivo* *In vitro*	20 mg/kg, i.g., for 7 days 10, 100 μM for 24 h	The C57BL/6 mice model of neuroinflammation was established by intraperitoneal injection of LPS. LPS-induced BV2 cells inflammatory model	Reduces TLR4 protein expression; Inhibits IKK and p65 phosphorylation	Rehman et al. (2018)
Phyllanthaceae	Phyllanthus amarus Schumach. & Thonn. Phyllanthin	*In vitro*	1.56–25 μM for 24 h	The human myeloid leukemia cells (U937) were induced to differentiate to obtain macrophage-like phenotype by the addition of PMA to the cells	Reduces TLR4, MyD88, IKK-α/β, p65 protein expression; Inhibits IKK-α/β, p65 phosphorylation; Inhibits IκB-α degradation	Harikrishnan et al. (2018)
Cucurbitaceae	Cucurbita moschata Duchesne Dehydrodiconiferyl alcohol	*In vivo* *In vitro*	100, 300 mg/kg, i.p., daily, for 7 days 10–80 μM for 24 h	Colitis was induced in C57BL/6 mice by the administration of 3.5% DSS dissolved in drinking water Bone marrow cells were prepared and differentiated into macrophages using M-CSF (*In vitro* model)	Reduces IKK-β protein expression; Inhibits IKK-β phosphorylation; Inhibits IκB-α degradation	Lee et al. (2015)
Oleaceae	Forsythia suspensa (Thunb.) Vahl Phillygenin	*In vitro*	6.25–200 μg/mL for 24 h	LPS-induced LX2 cells (hepatic stellate cells from the human liver that have been immobilized) inflammatory mode	Reduces TLR4, MyD88, TAK1, IKK-β, p65 protein expression; Inhibits IκB-α and p65 phosphorylation; Inhibits IκB-α degradation	Hu et al. (2020)
Myristicaceae	Myristica fragrans Houtt. Myrislignan	*In vitro*	6.25–50 μg/mL for 24 h	LPS-induced RAW264.7 inflammatory mode	Inhibits IκB-α ubiquitination; Inhibits p65 phosphorylation	Jin et al. (2012)
Cucurbitaceae	*Siraitia grosvenorii* (Swingle) C.Jeffrey ex A.M.Lu and Zhi Y.Zhang Mogroside V	*In vivo* *In vitro*	50 mg/kg, i.g., daily, for 32 days 100 μg/mL for 2 h	The Balb/c mice model of asthmatic was established by intraperitoneal injection of OVA. LPS-induced RAW264.7 inflammatory model	Reduces p65 protein expression; Inhibits p65 phosphorylation	Han et al. (2024)
Fabaceae	Glycyrrhiza uralensis Fisch. Glycyrrhetinic acid	*In vivo* *In vitro*	20 mg/kg, i.p., for 6 days 10–1,000 μg/mL for 6 h	The C57BL/6 mice model of MHV infection was established by intraperitoneal injection of plaque forming unit (PFU) of MHV-A59 Murine hepatitis virus (MHV) infection model	Reduces TLR4 expression	Shi et al. (2020)
Asparagaceae	*Anemarrhena asphodeloides* Bunge Timosaponin B-II	*In vivo*	20, 40 mg/kg, i.p., for 15 min	The Balb/C mice mode of acute lung injury was established by intratracheal injection of LPS.	Reduces TLR4, MyD88, p65 protein expression	Zhang et al. (2015b)
Araliaceae	Kalopanax pictus (Thunb.) Nakai Kalopanaxsaponin A	*In vivo* *In vitro*	10, 20 mg/kg, i.g., daily, for 3 days 50 ng/mL for 1 h	The ICR mice mode of Colitis was established by intrarectal administration of TNBS. Peritoneal macrophages from male ICR mice were stimulated with LPS or peptidoglycan	Reduces IRAK1, IκB-β protein expression; Inhibits IKK-β, p65 phosphorylation; Inhibits IκB-α degradation	Joh and Kim (2011)
Fabaceae	Glycine max (L.) Merr. Soyasaponin I	*In vivo* *In vitro*	10, 20 mg/kg, i.g., daily, for 5 days 2–20 μM for 20 h	The ICR mice mode of Colitis was established by intrarectal administration of TNBS. LPS-stimulated peritoneal macrophages from male C57BL/6 mice	Inhibits IκB-α phosphorylation; Prevents p65 nuclear translocation; Inhibits IκB-α degradation	Lee et al. (2010a)
Asparagaceae	Terauchia anemarrhenifolia Nakai Timosaponin AIII	*In vivo* *In vitro*	5, 10 mg/kg, i.g., daily, for 3 days 2–10 μM for 20 h	The C57BL/6 mice mode of Colitis was established by intrarectal administration of TNBS. LPS-stimulated peritoneal macrophages from male C57BL/6 mice	Reduces IRAK1, TAK1 potein expression; Inhibits IRAK1,TAK 1, IκB-α and p65 phosphorylation; Inhibits IκB-α degradation	Lim et al. (2015)
Araliaceae	Panax notoginseng (Burkill) F.H.Chen Notoginsenoside R1	*In vivo* *In vitro*	20 mg/kg, i.p., daily, for 8 weeks 5–200 μM for 24 h	The TNF-Tg mice established the RA model and were bred as heterozygotes on a C57BL/6 background, then Near infrared-indocyanine green (NIR-ICG) was injected into footpads Mouse Primary Lymphatic Endothelial Cells/C57-6,092 were stimulated by TNF-α and NG-R1	Reduces p65、 IKKα/β phosphorylation	Jiao et al. (2021)
Araliaceae	Panax ginseng C.A.Mey. Ginsenoside Rg5	*In vivo* *In vitro*	2.5, 5 mg/kg, i.p., for 1 h 5, 10 μM for 20 h	The C57BL/6 mice model of acute lung injury was established by intratracheal injection of LPS. Isolation of alveolar macrophages from alveolar lavage fluid	Reduces IRAK1/4 protein expression; Inhibits IRAK1, IKK-β, p65 phosphorylation	Kim et al. (2012)
Theaceae	Camellia sinensis (L.) Kuntze Catechins	*In vitro*	10 μg/L for 12, 24, 48 h	Human dental pulp cells were isolated from healthy permanent teeth	Inhibits p65 phosphorylation; Prevents p65 nuclear translocation	Wang et al. (2020)
—	— Resveratrol	*In vivo* *In vitro*	50, 100 mg/kg, i.g., daily, for 6 days 12.5–50 μM for 36 h	The Balb/c mice model of acute T. gondii infection was established by intraperitoneally (i.p.) injected with tachyzoites of T. gondii RH Strain NCTC 1469 cells were infected with T. gondii at the ratio of tachyzoite: cells = 5 : 1 for 4 h	Reduces TLR4 MyD88 protein expression; Prevents p65 nuclear translocation; Inhibits IκB-α degradation	Lu et al. (2021)
Asphodelaceae	*Aloe vera* (L.) Burm.f Aloin	*In vivo*	30 mg/kg, i.g., for 8 weeks	The C57BL/B6 mice model of neurodegenerative diseases was established by subcutaneous injection D-gal	Reduces p65 protein expression	Zhong et al. (2019)
Asteraceae	Inula britannica L. Eupatolide	*In vitro*	0.1–10 μM for 0.5 h	LPS-induced RAW264.7 inflammatory model	Reduces IκB-α and p65 protein expression; Inhibits IκB-α、IKK-α/β and p65 phosphorylation; Inhibits TRAF6 ubiquitination	Lee et al. (2010b)
Ranunculaceae	*Coptis chinensis* Franch. coptisine	*In vivo*	150 mg/kg, i.g., daily, for 12 weeks	The C57BL/6J mice model of atherosclerosis (AS) was established by gavage of 1.25% cholesterol and 21% fat orally daily for 12 weeks	Reduces p65 mRNA expression; Reduces IκB-α and p65 protein expression; Prevents p65 nuclear translocation;	Feng et al. (2017)
Gentianaceae	Gentiana cruciata *L* Gentiopicroside	*In vivo* *In vitro*	50 mg/kg, i.p., for 30 min 1,000 μg/mL	The C57BL/6 mice model of sepsis was established by intraperitoneally injected of LPS. LPS and IFN-γinduced primary bone marrow-derived macrophages (BMMs) or peritoneal macrophages (PEMs) inflammatory model	Inhibits IKK-α/β and p65 phosphorylation; Prevents p65 nuclear translocation; Inhibits IκB-α degradation	Wang et al. (2019)
Brassicaceae	*Isatis tinctoria* L Isatidis folium water extract (WIF)	*In vivo* *In vitro*	100, 200 mg/kg, i.g 50, 100, and 200 μg/mL for 1 h	The Balb/c mice model of atopic dermatitis was established by intraperitoneally injected of 2,4-dinitrochlorobenzene (DNCB) TNF-α/IFN-γinduced HaCaT cells inflammatory model	Prevents p65 nuclear translocation	Min et al. (2023)
Caprifoliaceae	*Lonicera japonica* Thunb *Lonicera japonica* Thunb extrate (LTE) and luteolin	*In vivo* *In vitro*	LTE 1.75 g/kg or Lut 18, 35, 70 μmol/kg, i.g., for 7 days LTE 10 ug/mLor Lut 10 µM for 24 h	The C57BL/6 mice model of ALI was established by intraperitoneal injection of LPS. LPS-induced BEAS-2B cells inflammatory model	Reduces MyD88 and IκB-α protein expression; Inhibits p65 phosphorylation	Jia et al. (2023)
Ranunculaceae	*Coptis chinensis* Franch Berberine	*In vivo* *In vitro*	40 mg/kg i.g., daily, for 1 or 4 weeks 5 μM for 24 h	The C57BL/6 mice model of acute inflammation was established by intraperitoneally injected of LPS. The C57BL/6 mice model of chronic inflammation was established by fed a high fat diet (20 kcal% carbohy-drates, 20 kcal% protein and 60 kcal% fat) LPS-induced RAW264.7 and BMDM cells inflamma-tory model	Reduce p65 acetylation; Prevents p65 nuclear translocation	Zhang et al. (2023)
—	— Resveratrol	*In vivo* *In vitro*	20 mg/kg i.p., daily, for 6 weeks 0, 2.5, 5, 7.5, 10 μM for 2 and 6 h	MRL/lpr mice (Spontaneous lupus erythematosus like mouse model) —	Reduce p65 acetylation; Prevents p65 nuclear translocation	Jhou et al. (2017)

## 6 Discussion

The TLR4/NF-κB pathway plays a crucial regulatory role in the field of inflammation. As a member of the nuclear transcription factor family, NF-κB protein can regulate the transcription of various genes, inducing the transcription of genes encoding inflammatory mediators such as cytokines, chemokines, and adhesion molecules. Activation of the TLR4/NF-κB signaling pathway is primarily initiated by TLR4 recognizing pathogen molecules like LPS as ligands, through two signaling pathways dependent on MyD88 and TRIF, activating the downstream TRAF6 as an E3 ubiquitin ligase to form free K63 ubiquitin chains. Upon receiving the K63 ubiquitin chain signal, the downstream IKK complex promotes IκB phosphorylation and degradation, releasing NF-κB p65, initiating the nuclear translocation of NF-κB, where it exerts its role as a transcription factor, regulating the transcription of various genes such as TNF-α, IL-1, IL-6. The release of these inflammation-related factors triggers inflammatory responses, leading to processes like vasodilation, leukocyte infiltration, and tissue damage in inflammatory pathologies.

**FIGURE 4:**
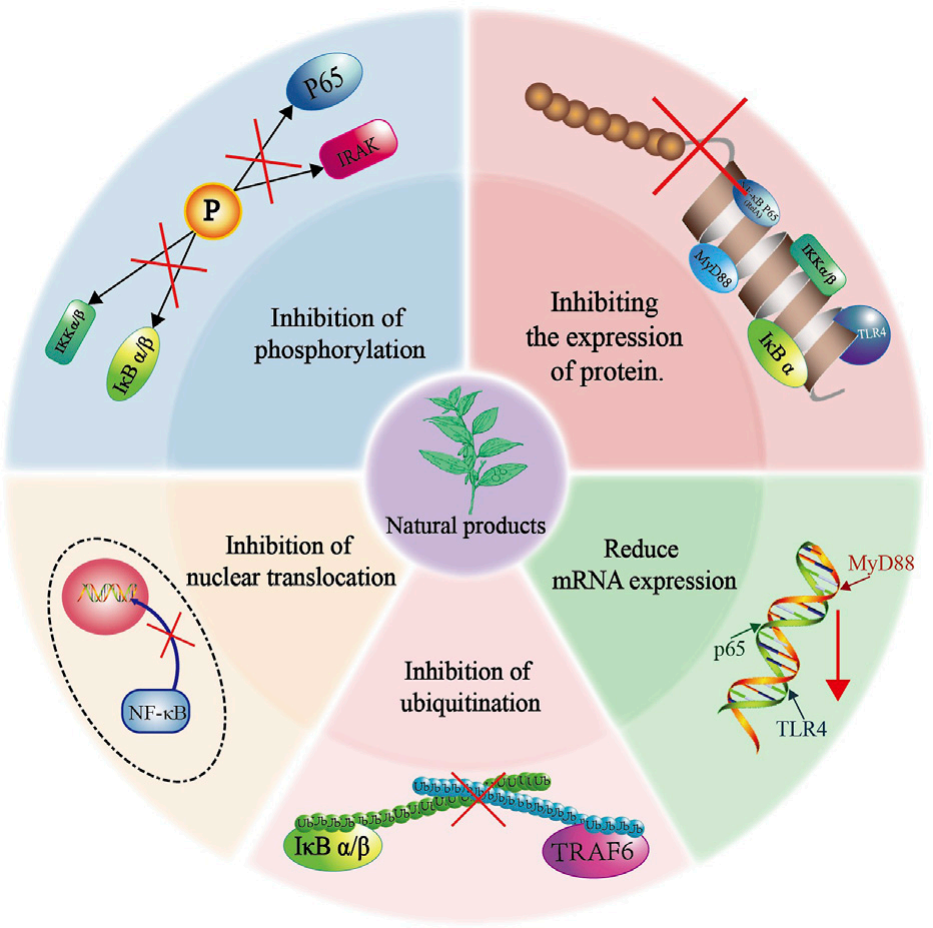
Mechanisms of Natural products’ anti-inflammatory effects based on the NF-κB pathway.

Natural product compounds demonstrate significant biological activities and functional diversity by influencing multiple targets within the TLR4/NF-κB pathway. They effectively inhibit the expression of proteins and mRNA, suppress the phosphorylation and ubiquitination of key proteins, and hinder the translocation of p65 into the nucleus, thereby exhibiting anti-inflammatory properties (Figure 4). In most current studies, it has been shown that Natural products can interact with multiple protein targets, but it has become difficult to identify the most biologically active true target (Rix and Superti-Furga, 2009; Klessig et al., 2016). With the advancement of molecular biology and the arrival of the post-genomic era, more and more research on Natural products mechanisms is being combined with chemical proteomics techniques. This comprehensive method of searching and identifying multiple protein targets in active small molecules can effectively identify the true targets. Subsequent validation of the screened targets in omics through molecular biology and pharmacological experiments can greatly improve the scientific validity and credibility of the research (Wang S. et al., 2018).

In addition, compared to traditional chemically synthesized drugs, the efficacy of Natural products may be affected by factors such as plant origin, growth environment, and collection time, resulting in unstable efficacy and difficulty in ensuring consistency with each use. Therefore, by modifying the chemical structure, the bioavailability, metabolic pathways, and targeting of compounds can be altered to enhance their pharmacological effects. And develop drug delivery systems targeting natural products, such as microspheres, nanoparticles, liposomes, etc., which can improve their release rate and bioavailability *in vivo*, and enhance the stability of drug efficacy. Therefore, it is essential to focus on interdisciplinary collaboration, integrating disciplines such as pharmacology, pharmacy, and molecular materials science, which can contribute to the significant development of research on Natural products.

Collectively, NF-κB, as a central regulatory factor in the treatment and intervention of inflammatory diseases, will provide valuable insights for the development of new anti-inflammatory natural plant-based drugs with better efficacy and safety by introducing high-throughput omics techniques and emphasizing interdisciplinary collaboration to deeply study the activation mechanism of the NF-κB pathway by natural products.

## References

[B1] Adhikari A. Xu M. Chen Z. J. (2007). Ubiquitin-mediated activation of TAK1 and IKK. Oncogene 26, 3214–3226. 10.1038/sj.onc.1210413 17496917

[B2] Akashi S. Shimazu R. Ogata H. Nagai Y. Takeda K. Kimoto M. (2000). Cutting edge: cell surface expression and lipopolysaccharide signaling via the toll-like receptor 4-MD-2 complex on mouse peritoneal macrophages. J. Immunol. 164, 3471–3475. 10.4049/jimmunol.164.7.3471 10725698

[B3] Akira S. (2003). Toll-like receptor signaling. J. Biol. Chem. 278, 38105–38108. 10.1074/jbc.R300028200 12893815

[B4] Alarcón-Vila C. Baroja-Mazo A. De Torre-Minguela C. Martínez C. M. Martínez-García J. J. Martínez-Banaclocha H. (2020). CD14 release induced by P2X7 receptor restricts inflammation and increases survival during sepsis. Elife 9, e60849. 10.7554/eLife.60849 33135636 PMC7690950

[B5] Aletaha D. Smolen J. S. (2018). Diagnosis and management of rheumatoid arthritis: a review. JAMA 320, 1360.1360–1372. 10.1001/jama.2018.13103 30285183

[B6] Álvarez-Martínez F. J. Barrajón-Catalán E. Micol V. (2020). Tackling antibiotic resistance with compounds of natural origin: a comprehensive review. Biomedicines 8, 405. 10.3390/biomedicines8100405 33050619 PMC7601869

[B7] Anderberg S. B. Luther T. Frithiof R. (2017). Physiological aspects of Toll-like receptor 4 activation in sepsis-induced acute kidney injury. Acta Physiol. (Oxf) 219, 573–588. 10.1111/apha.12798 27602552 PMC5324638

[B8] Baur J. A. Sinclair D. A. (2006). Therapeutic potential of resveratrol: the *in vivo* evidence. Nat. Rev. Drug Discov. 5, 493–506. 10.1038/nrd2060 16732220

[B9] Bonham K. S. Orzalli M. H. Hayashi K. Wolf A. I. Glanemann C. Weninger W. (2014). A promiscuous lipid-binding protein diversifies the subcellular sites of toll-like receptor signal transduction. Cell 156, 705–716. 10.1016/j.cell.2014.01.019 24529375 PMC3951743

[B10] Cai C. Tang Y.-D. Zhai J. Zheng C. (2022). The RING finger protein family in health and disease. Signal Transduct. Target. Ther. 7, 300. 10.1038/s41392-022-01152-2 36042206 PMC9424811

[B12] Chen W. Li Z. Bai L. Lin Y. (2011). NF-kappaB in lung cancer, a carcinogenesis mediator and a prevention and therapy target. FBL 16, 1172–1185. 10.2741/3782 21196225 PMC3032584

[B13] Cildir G. Low K. C. Tergaonkar V. (2016). Noncanonical NF-κB signaling in health and disease. Trends Mol. Med. 22, 414–429. 10.1016/j.molmed.2016.03.002 27068135

[B14] Deng L. Wang C. Spencer E. Yang L. Braun A. You J. (2000). Activation of the IkappaB kinase complex by TRAF6 requires a dimeric ubiquitin-conjugating enzyme complex and a unique polyubiquitin chain. Cell 103, 351–361. 10.1016/s0092-8674(00)00126-4 11057907

[B15] Di Lorenzo F. Duda K. A. Lanzetta R. Silipo A. De Castro C. Molinaro A. (2022). A journey from structure to function of bacterial lipopolysaccharides. Chem. Rev. 122, 15767–15821. 10.1021/acs.chemrev.0c01321 34286971

[B16] Dong Y. Zhang T. Li J. Deng H. Song Y. Zhai D. (2014). Oridonin inhibits tumor growth and metastasis through anti-angiogenesis by blocking the Notch signaling. PLoS One 9, e113830. 10.1371/journal.pone.0113830 25485753 PMC4259472

[B17] Doyle S. L. O'neill L. A. (2006). Toll-like receptors: from the discovery of NFkappaB to new insights into transcriptional regulations in innate immunity. Biochem. Pharmacol. 72, 1102–1113. 10.1016/j.bcp.2006.07.010 16930560

[B18] Fan X. Zhu J.-Y. Sun Y. Luo L. Yan J. Yang X. (2017). Evodiamine inhibits zymosan-induced inflammation *in vitro* and *in vivo*: inactivation of NF-κB by inhibiting IκBα phosphorylation. Inflammation 40, 1012–1027. 10.1007/s10753-017-0546-0 28337636

[B19] Fan Z. Yao J. Li Y. Hu X. Shao H. Tian X. (2015). Anti-inflammatory and antioxidant effects of curcumin on acute lung injury in a rodent model of intestinal ischemia reperfusion by inhibiting the pathway of NF-Kb. Int. J. Clin. Exp. Pathol. 8, 3451–3459. 26097529 PMC4466916

[B20] Fang S. Zhang B. Xiang W. Zheng L. Wang X. Li S. (2024). Natural products in osteoarthritis treatment: bridging basic research to clinical applications. Chin. Med. 19, 25. 10.1186/s13020-024-00899-w 38360724 PMC10870578

[B21] Feng M. Kong S. Z. Wang Z. X. He K. Zou Z. Y. Hu Y. R. (2017). The protective effect of coptisine on experimental atherosclerosis ApoE(-/-) mice is mediated by MAPK/NF-κB-dependent pathway. Biomed. Pharmacother. 93, 721–729. 10.1016/j.biopha.2017.07.002 28700976

[B22] Fitzgerald K. A. Kagan J. C. (2020). Toll-like receptors and the control of immunity. Cell 180, 1044–1066. 10.1016/j.cell.2020.02.041 32164908 PMC9358771

[B23] Fu Y.-J. Xu B. Huang S.-W. Luo X. Deng X.-L. Luo S. (2020). Baicalin prevents LPS-induced activation of TLR4/NF-κB p65 pathway and inflammation in mice via inhibiting the expression of CD14. Acta Pharmacol. Sin. 42, 88–96. 10.1038/s41401-020-0411-9 32457419 PMC7921675

[B24] Furman D. Campisi J. Verdin E. Carrera-Bastos P. Targ S. Franceschi C. (2019). Chronic inflammation in the etiology of disease across the life span. Nat. Med. 25, 1822–1832. 10.1038/s41591-019-0675-0 31806905 PMC7147972

[B25] Gallo L. H. Ko J. Donoghue D. J. (2017). The importance of regulatory ubiquitination in cancer and metastasis. Cell Cycle 16, 634–648. 10.1080/15384101.2017.1288326 28166483 PMC5397262

[B26] Gan F. Liu Q. Liu Y. Huang D. Pan C. Song S. (2018). Lycium barbarum polysaccharides improve CCl(4)-induced liver fibrosis, inflammatory response and TLRs/NF-kB signaling pathway expression in wistar rats. Life Sci. 192, 205–212. 10.1016/j.lfs.2017.11.047 29196051

[B27] Ghosh S. Hayden M. S. (2008). New regulators of NF-kappaB in inflammation. Nat. Rev. Immunol. 8, 837–848. 10.1038/nri2423 18927578

[B28] Gray P. Dunne A. Brikos C. Jefferies C. A. Doyle S. L. La O. N. (2016). MyD88 adapter-like (Mal) is phosphorylated by Bruton's tyrosine kinase during TLR2 and TLR4 signal transduction. J. Biol. Chem. 291, 26240. 10.1074/jbc.A116.508892 27941071 PMC5207091

[B29] Guo C. He L. Hu N. Zhao X. Gong L. Wang C. (2022). Aconiti Lateralis Radix Praeparata lipid-soluble alkaloids alleviates IL-1β-induced inflammation of human fibroblast-like synoviocytes in rheumatoid arthritis by inhibiting NF-κB and MAPKs signaling pathways and inducing apoptosis. Cytokine 151, 155809. 10.1016/j.cyto.2022.155809 35092909

[B30] Guo Y. Peng X. Liu F. Zhang Q. Ding L. Li G. (2024). Potential of natural products in inflammation: biological activities, structure-activity relationships, and mechanistic targets. Arch. Pharm. Res. 47, 377–409. 10.1007/s12272-024-01496-z 38739203

[B31] Häcker H. Karin M. (2006). Regulation and function of IKK and IKK-related kinases. Sci. STKE 2006, re13. 10.1126/stke.3572006re13 17047224

[B32] Haftcheshmeh S. M. Abedi M. Mashayekhi K. Mousavi M. J. Navashenaq J. G. Mohammadi A. (2022). Berberine as a natural modulator of inflammatory signaling pathways in the immune system: focus on NF‐κB, JAK/STAT, and MAPK signaling pathways. Phytotherapy Res. 36, 1216–1230. 10.1002/ptr.7407 35142403

[B33] Han M. Liu H. Liu G. Li X. Zhou L. Liu Y. (2024). Mogroside V alleviates inflammation response by modulating miR-21-5P/SPRY1 axis. Food Funct. 15, 1909–1922. 10.1039/d3fo01901b 38258992

[B34] Harikrishnan H. Jantan I. Haque M. A. Kumolosasi E. (2018). Phyllanthin from Phyllanthus amarus inhibits LPS-induced proinflammatory responses in U937 macrophages via downregulation of NF-κB/MAPK/PI3K-Akt signaling pathways. Phytotherapy Res. 32, 2510–2519. 10.1002/ptr.6190 30238535

[B35] Hayden M. S. Ghosh S. (2008). Shared principles in NF-kappaB signaling. Cell 132, 344–362. 10.1016/j.cell.2008.01.020 18267068

[B36] Hoffmann A. Natoli G. Ghosh G. (2006). Transcriptional regulation via the NF-kappaB signaling module. Oncogene 25, 6706–6716. 10.1038/sj.onc.1209933 17072323

[B37] Hou J. J. Zhang Z. J. Wu W. Y. He Q. Q. Zhang T. Q. Liu Y. W. (2022). Mass spectrometry imaging: new eyes on natural products for drug research and development. Acta Pharmacol. Sin. 43, 3096–3111. 10.1038/s41401-022-00990-8 36229602 PMC9712638

[B38] Hu N. Wang C. Dai X. Zhou M. Gong L. Yu L. (2020). Phillygenin inhibits LPS-induced activation and inflammation of LX2 cells by TLR4/MyD88/NF-κB signaling pathway. J. Ethnopharmacol. 248, 112361. 10.1016/j.jep.2019.112361 31683033

[B39] Huang M. Tan Y.-Q. Luo J. Shen J.-Y. (2018). Antimicrobial resistance of Chinese herbal medicine. Chin. J. Exp. Traditional Med. Formulae 24, 218–224. 10.13422/j.cnki.syfjx.20182336

[B40] Israël A. (2010). The IKK complex, a central regulator of NF-kappaB activation. Cold Spring Harb. Perspect. Biol. 2, a000158. 10.1101/cshperspect.a000158 20300203 PMC2829958

[B41] Itoh H. Inoue M. (2019). Comprehensive structure-activity relationship studies of macrocyclic natural products enabled by their total syntheses. Chem. Rev. 119, 10002–10031. 10.1021/acs.chemrev.9b00063 30945851

[B42] Iwai K. (2012). Diverse ubiquitin signaling in NF-κB activation. Trends Cell Biol. 22, 355–364. 10.1016/j.tcb.2012.04.001 22543051

[B43] Jeong J.-W. Lee H. H. Han M. H. Kim G.-Y. Kim W.-J. Choi Y. H. (2014). Anti-inflammatory effects of genistein via suppression of the toll-like receptor 4-mediated signaling pathway in lipopolysaccharide-stimulated BV2 microglia. Chemico-biological Interact. 212, 30–39. 10.1016/j.cbi.2014.01.012 24491678

[B44] Jhou J. P. Chen S. J. Huang H. Y. Lin W. W. Huang D. Y. Tzeng S. J. (2017). Upregulation of FcγRIIB by resveratrol via NF-κB activation reduces B-cell numbers and ameliorates lupus. Exp. Mol. Med. 49, e381. 10.1038/emm.2017.144 28960214 PMC5628277

[B45] Jia Q. Wen J. Yang Q. Liu S. Zhang J. Wang T. (2023). Lonicera japonica Thunb extract ameliorates lipopolysaccharide-induced acute lung injury associated with luteolin-mediated suppression of NF-κB signaling pathway. J. Inflamm. (Lond) 20, 44. 10.1186/s12950-023-00372-9 38115057 PMC10729360

[B46] Jiang Q. Yi M. Guo Q. Wang C. Wang H. Meng S. (2015). Protective effects of polydatin on lipopolysaccharide-induced acute lung injury through TLR4-MyD88-NF-κB pathway. Int. Immunopharmacol. 29, 370–376. 10.1016/j.intimp.2015.10.027 26507165

[B47] Jiang Z. Mak T. W. Sen G. Li X. (2004). Toll-like receptor 3-mediated activation of NF-kappaB and IRF3 diverges at Toll-IL-1 receptor domain-containing adapter inducing IFN-beta. Proc. Natl. Acad. Sci. U. S. A. 101, 3533–3538. 10.1073/pnas.0308496101 14982987 PMC373497

[B48] Jiao D. Liu Y. Hou T. Xu H. Wang X. Shi Q. (2021). Notoginsenoside R1 (NG-R1) promoted lymphatic drainage function to ameliorating rheumatoid arthritis in TNF-tg mice by suppressing NF-κB signaling pathway. Front. Pharmacol. 12, 730579. 10.3389/fphar.2021.730579 35280253 PMC8909130

[B49] Jin C.-Y. Lee J.-D. Park C. Choi Y. H. Kim G.-Y. (2007). Curcumin attenuates the release of pro-inflammatory cytokines in lipopolysaccharide-stimulated BV2 microglia. Acta Pharmacol. Sin. 28, 1645–1651. 10.1111/j.1745-7254.2007.00651.x 17883952

[B50] Jin H. Zhu Z.-G. Yu P.-J. Wang G.-F. Zhang J.-Y. Li J.-R. (2012). Myrislignan attenuates lipopolysaccharide-induced inflammation reaction in murine macrophage cells through inhibition of NF-κB signalling pathway activation. Phytotherapy Res. 26, 1320–1326. 10.1002/ptr.3707 22294521

[B51] Jin L. Williamson A. Banerjee S. Philipp I. Rape M. (2008). Mechanism of ubiquitin-chain formation by the human anaphase-promoting complex. Cell 133, 653–665. 10.1016/j.cell.2008.04.012 18485873 PMC2696189

[B52] Joh E.-H. Kim D.-H. (2011). Kalopanaxsaponin A ameliorates experimental colitis in mice by inhibiting IRAK-1 activation in the NF-κB and MAPK pathways. Br. J. Pharmacol. 162 (0), 1731–1742. 10.1111/j.1476-5381.2010.01195.x 21198552 PMC3081117

[B53] Kagan J. C. (2012). Signaling organelles of the innate immune system. Cell 151, 1168–1178. 10.1016/j.cell.2012.11.011 23217704 PMC3523748

[B54] Kagan J. C. Su T. Horng T. Chow A. Akira S. Medzhitov R. (2008). TRAM couples endocytosis of Toll-like receptor 4 to the induction of interferon-beta. Nat. Immunol. 9, 361–368. 10.1038/ni1569 18297073 PMC4112825

[B55] Kavanaugh A. Wells A. F. (2014). Benefits and risks of low-dose glucocorticoid treatment in the patient with rheumatoid arthritis. Rheumatology 53, 1742–1751. 10.1093/rheumatology/keu135 24729402 PMC4165844

[B56] Kelley S. L. Lukk T. Nair S. K. Tapping R. I. (2013). The crystal structure of human soluble CD14 reveals a bent solenoid with a hydrophobic amino-terminal pocket. J. Immunol. 190, 1304–1311. 10.4049/jimmunol.1202446 23264655 PMC3552104

[B57] Kim J. I. Lee C. J. Jin M. S. Lee C. H. Paik S. G. Lee H. (2005). Crystal structure of CD14 and its implications for lipopolysaccharide signaling. J. Biol. Chem. 280, 11347–11351. 10.1074/jbc.M414607200 15644310

[B58] Kim T. W. Joh E. H. Kim B. Kim D. H. (2012). Ginsenoside Rg5 ameliorates lung inflammation in mice by inhibiting the binding of LPS to toll-like receptor-4 on macrophages. Int. Immunopharmacol. 12, 110–116. 10.1016/j.intimp.2011.10.023 22107725

[B59] Klessig D. F. Tian M. Choi H. W. (2016). Multiple targets of salicylic acid and its derivatives in plants and animals. Front. Immunol. 7, 206. 10.3389/fimmu.2016.00206 27303403 PMC4880560

[B60] Kobayashi M. Saitoh S. Tanimura N. Takahashi K. Kawasaki K. Nishijima M. (2006). Regulatory roles for MD-2 and TLR4 in ligand-induced receptor clustering. J. Immunol. 176, 6211–6218. 10.4049/jimmunol.176.10.6211 16670331

[B61] Kotas M. E. Medzhitov R. (2015). Homeostasis, inflammation, and disease susceptibility. Cell 160, 816–827. 10.1016/j.cell.2015.02.010 25723161 PMC4369762

[B62] Le K. Song Z. Deng J. Peng X. Zhang J. Wang L. (2020). Quercetin alleviates neonatal hypoxic-ischemic brain injury by inhibiting microglia-derived oxidative stress and TLR4-mediated inflammation. Inflamm. Res. 69, 1201–1213. 10.1007/s00011-020-01402-5 32944799

[B63] Lee I.-A. Park Y.-J. Yeo H.-K. Han M. J. Kim D.-H. (2010a). Soyasaponin I attenuates TNBS-Induced colitis in mice by inhibiting NF-κB pathway. J. Agric. Food Chem. 58, 10929–10934. 10.1021/jf102296y 20923188

[B64] Lee J. Choi J. Kim S. (2015). Effective suppression of pro-inflammatory molecules by DHCA via IKK-NF-κB pathway, *in vitro* and *in vivo* . Br. J. Pharmacol. 172 (0), 3353–3369. 10.1111/bph.13137 25802070 PMC4500371

[B65] Lee J. Tae N. Lee J. J. Kim T. Lee J. H. (2010b). Eupatolide inhibits lipopolysaccharide-induced COX-2 and iNOS expression in RAW264.7 cells by inducing proteasomal degradation of TRAF6. Eur. J. Pharmacol. 636, 173–180. 10.1016/j.ejphar.2010.03.021 20353767

[B66] Leung E. Weil D. E. Raviglione M. Nakatani H. World Health Organization World Health Day Antimicrobial Resistance Technical Working, G (2011). The WHO policy package to combat antimicrobial resistance. Bull. World Health Organ 89, 390–392. 10.2471/BLT.11.088435 21556308 PMC3089396

[B67] Li D. Chen J. Ye J. Zhai X. Song J. Jiang C. (2017). Anti-inflammatory effect of the six compounds isolated from Nauclea officinalis Pierrc ex Pitard, and molecular mechanism of strictosamide via suppressing the NF-κB and MAPK signaling pathway in LPS-induced RAW 264.7 macrophages. J. Ethnopharmacol. 196, 66–74. 10.1016/j.jep.2016.12.007 27989509

[B68] Li H. Fan C. Lu H. Feng C. He P. Yang X. (2020). Protective role of berberine on ulcerative colitis through modulating enteric glial cells-intestinal epithelial cells-immune cells interactions. Acta Pharm. Sin. B 10, 447–461. 10.1016/j.apsb.2019.08.006 32140391 PMC7049614

[B69] Li H. Xu J. Li X. Hu Y. Liao Y. Zhou W. (2021). Anti-inflammatory activity of psoralen in human periodontal ligament cells via estrogen receptor signaling pathway. Sci. Rep. 11, 8754. 10.1038/s41598-021-85145-1 33888745 PMC8062431

[B70] Li J. Bao L. Zha D. Zhang L. Gao P. Zhang J. (2018). Oridonin protects against the inflammatory response in diabetic nephropathy by inhibiting the TLR4/p38-MAPK and TLR4/NF-κB signaling pathways. Int. Immunopharmacol. 55, 9–19. 10.1016/j.intimp.2017.11.040 29207360

[B71] Liao W. He X. Yi Z. Xiang W. Ding Y. (2018). Chelidonine suppresses LPS-Induced production of inflammatory mediators through the inhibitory of the TLR4/NF-κB signaling pathway in RAW264.7 macrophages. Biomed. and Pharmacother. 107, 1151–1159. 10.1016/j.biopha.2018.08.094 30257328

[B72] Lim S. M. Jeong J. J. Kang G. D. Kim K. A. Choi H. S. Kim D. H. (2015). Timosaponin AIII and its metabolite sarsasapogenin ameliorate colitis in mice by inhibiting NF-κB and MAPK activation and restoring Th17/Treg cell balance. Int. Immunopharmacol. 25, 493–503. 10.1016/j.intimp.2015.02.016 25698557

[B73] Liu C. Liu S. Xiong L. Zhang L. Li X. Cao X. (2021). Genistein-3'-sodium sulfonate attenuates neuroinflammation in stroke rats by down-regulating microglial M1 polarization through α7nAChR-NF-κB signaling pathway. Int. J. Biol. Sci. 17, 1088–1100. 10.7150/ijbs.56800 33867831 PMC8040300

[B74] Liu Q. Ma Y. Alhussein M. Zhang Y. Peng L. (2016). Green data center with IoT sensing and cloud-assisted smart temperature control system. Comput. Netw. 101, 104–112. 10.1016/j.comnet.2015.11.024

[B75] Liu X. Li M. Tan S. Wang C. Fan S. Huang C. (2017). Harmine is an inflammatory inhibitor through the suppression of NF-κB signaling. Biochem. biophysical Res. Commun. 489, 332–338. 10.1016/j.bbrc.2017.05.126 28551404

[B76] Liu Y. Yin H. Zhao M. Lu Q. (2014). TLR2 and TLR4 in autoimmune diseases: a comprehensive review. Clin. Rev. Allergy and Immunol. 47, 136–147. 10.1007/s12016-013-8402-y 24352680

[B77] Lu J.-M. Jin G.-N. Lu Y.-N. Zhao X.-D. Lan H.-W. Mu S.-R. (2021). Resveratrol modulates Toxoplasma gondii infection induced liver injury by intervening in the HMGB1/TLR4/NF-κB signaling pathway. Eur. J. Pharmacol. 910, 174497. 10.1016/j.ejphar.2021.174497 34508751

[B78] Lu M. Zhang Q. Chen K. Xu W. Xiang X. Xia S. (2017a). The regulatory effect of oxymatrine on the TLR4/MyD88/NF-κB signaling pathway in lipopolysaccharide-induced MS1 cells. Phytomedicine 36, 153–159. 10.1016/j.phymed.2017.10.001 29157809

[B79] Lu X. Pu Y. Kong W. Tang X. Zhou J. Gou H. (2017b). Antidesmone, a unique tetrahydroquinoline alkaloid, prevents acute lung injury via regulating MAPK and NF-κB activities. Int. Immunopharmacol. 45, 34–42. 10.1016/j.intimp.2017.01.026 28157559

[B80] Mathiesen O. Wetterslev J. Kontinen V. K. Pommergaard H. C. Nikolajsen L. Rosenberg J. (2014). Adverse effects of perioperative paracetamol, NSAIDs, glucocorticoids, gabapentinoids and their combinations: a topical review. Acta Anaesthesiol. Scand. 58, 1182–1198. 10.1111/aas.12380 25116762

[B81] Mendes K. L. Lelis D. F. Santos S. H. S. (2017). Nuclear sirtuins and inflammatory signaling pathways. Cytokine Growth Factor Rev. 38, 98–105. 10.1016/j.cytogfr.2017.11.001 29132743

[B82] Meng M. Wang L. Yao Y. Lin D. Wang C. Yao J. (2023). Ganoderma lucidum polysaccharide peptide (GLPP) attenuates rheumatic arthritis in rats through inactivating NF-κB and MAPK signaling pathways. Phytomedicine 119, 155010. 10.1016/j.phymed.2023.155010 37586160

[B83] Min G. Y. Kim T. I. Kim J. H. Cho W. K. Yang J. H. Ma J. Y. (2023). Anti-atopic effect of isatidis folium water extract in TNF-α/IFN-γ-Induced HaCaT cells and DNCB-induced atopic dermatitis mouse model. Molecules 28, 3960. 10.3390/molecules28093960 37175371 PMC10180365

[B84] Muhammad T. Ikram M. Ullah R. Rehman S. U. Kim M. O. (2019). Hesperetin, a citrus flavonoid, attenuates LPS-induced neuroinflammation, apoptosis and memory impairments by modulating TLR4/NF-κB signaling. Nutrients 11, 648. 10.3390/nu11030648 30884890 PMC6471991

[B85] Newman D. J. Cragg G. M. (2007). Natural products as sources of new drugs over the last 25 years. J. Nat. Prod. 70, 461–477. 10.1021/np068054v 17309302

[B86] Newton K. Matsumoto M. L. Wertz I. E. Kirkpatrick D. S. Lill J. R. Tan J. (2008). Ubiquitin chain editing revealed by polyubiquitin linkage-specific antibodies. Cell 134, 668–678. 10.1016/j.cell.2008.07.039 18724939

[B87] Ng J. C. Yeomans N. D. (2018). Helicobacter pylori infection and the risk of upper gastrointestinal bleeding in low dose aspirin users: systematic review and meta-analysis. Med. J. Aust. 209, 306–311. 10.5694/mja17.01274 30257623

[B88] Nielsen A. J. Mcnulty J. (2019). Polyphenolic natural products and natural product-inspired steroidal mimics as aromatase inhibitors. Med. Res. Rev. 39, 1274–1293. 10.1002/med.21536 30171625

[B89] Niu Y. Dong Q. Li R. (2017). Matrine regulates Th1/Th2 cytokine responses in rheumatoid arthritis by attenuating the NF-κB signaling. Cell Biol. Int. 41, 611–621. 10.1002/cbin.10763 28295853

[B91] O'neill L. A. Bowie A. G. (2007). The family of five: TIR-domain-containing adaptors in Toll-like receptor signalling. Nat. Rev. Immunol. 7, 353–364. 10.1038/nri2079 17457343

[B92] Park B. S. Lee J. O. (2013). Recognition of lipopolysaccharide pattern by TLR4 complexes. Exp. Mol. Med. 45, e66. 10.1038/emm.2013.97 24310172 PMC3880462

[B93] Pei H. Xue L. Tang M. Tang H. Kuang S. Wang L. (2020). Alkaloids from black pepper (Piper nigrum L.) exhibit anti-inflammatory activity in murine macrophages by inhibiting activation of NF-κB pathway. J. Agric. Food Chem. 68, 2406–2417. 10.1021/acs.jafc.9b07754 32031370

[B94] Peng Q. Liu H. Shi S. Li M. (2014). Lycium ruthenicum polysaccharide attenuates inflammation through inhibiting TLR4/NF-κB signaling pathway. Int. J. Biol. Macromol. 67, 330–335. 10.1016/j.ijbiomac.2014.03.023 24680899

[B95] Ran J. Ma C. Xu K. Xu L. He Y. Moqbel S. a.A. (2018). Schisandrin B ameliorated chondrocytes inflammation and osteoarthritis via suppression of NF-κB and MAPK signal pathways. Drug Des. Dev. Ther. 12, 1195–1204. 10.2147/dddt.S162014 PMC5953308 29785089

[B96] Raza S. S. Khan M. M. Ahmad A. Ashafaq M. Islam F. Wagner A. P. (2013). Neuroprotective effect of naringenin is mediated through suppression of NF-κB signaling pathway in experimental stroke. Neuroscience 230, 157–171. 10.1016/j.neuroscience.2012.10.041 23103795

[B97] Rehman S. U. Ali T. Alam S. I. Ullah R. Zeb A. Lee K. W. (2018). Ferulic acid rescues LPS-induced neurotoxicity via modulation of the TLR4 receptor in the mouse Hippocampus. Mol. Neurobiol. 56, 2774–2790. 10.1007/s12035-018-1280-9 30058023

[B98] Rim H. K. Cho W. Sung S. H. Lee K. T. (2012). Nodakenin suppresses lipopolysaccharide-induced inflammatory responses in macrophage cells by inhibiting tumor necrosis factor receptor-associated factor 6 and nuclear factor-κB pathways and protects mice from lethal endotoxin shock. J. Pharmacol. Exp. Ther. 342, 654–664. 10.1124/jpet.112.194613 22637723

[B99] Rix U. Superti-Furga G. (2009). Target profiling of small molecules by chemical proteomics. Nat. Chem. Biol. 5, 616–624. 10.1038/nchembio.216 19690537

[B100] Rodrigues T. Reker D. Schneider P. Schneider G. (2016). Counting on natural products for drug design. Nat. Chem. 8, 531–541. 10.1038/nchem.2479 27219696

[B101] Rowe D. C. Mcgettrick A. F. Latz E. Monks B. G. Gay N. J. Yamamoto M. (2006). The myristoylation of TRIF-related adaptor molecule is essential for Toll-like receptor 4 signal transduction. Proc. Natl. Acad. Sci. U. S. A. 103, 6299–6304. 10.1073/pnas.0510041103 16603631 PMC1458872

[B102] Ruland J. (2011). Return to homeostasis: downregulation of NF-κB responses. Nat. Immunol. 12, 709–714. 10.1038/ni.2055 21772279

[B103] Sato S. Sugiyama M. Yamamoto M. Watanabe Y. Kawai T. Takeda K. (2003). Toll/IL-1 receptor domain-containing adaptor inducing IFN-beta (TRIF) associates with TNF receptor-associated factor 6 and TANK-binding kinase 1, and activates two distinct transcription factors, NF-kappa B and IFN-regulatory factor-3, in the Toll-like receptor signaling. J. Immunol. 171, 4304–4310. 10.4049/jimmunol.171.8.4304 14530355

[B104] Sehnert B. Burkhardt H. Dubel S. Voll R. E. (2020). Cell-type targeted NF-kappaB inhibition for the treatment of inflammatory diseases. Cells 9, 1627. 10.3390/cells9071627 32640727 PMC7407293

[B105] Shen C.-Y. Xu X.-L. Yang L.-J. Jiang J.-G. (2019). Identification of narciclasine from Lycoris radiata (L'Her.) Herb. and its inhibitory effect on LPS-induced inflammatory responses in macrophages. Food Chem. Toxicol. 125, 605–613. 10.1016/j.fct.2019.02.003 30738987

[B106] Shi X. Yu L. Zhang Y. Liu Z. Zhang H. Zhang Y. (2020). Glycyrrhetinic acid alleviates hepatic inflammation injury in viral hepatitis disease via a HMGB1-TLR4 signaling pathway. Int. Immunopharmacol. 84, 106578. 10.1016/j.intimp.2020.106578 32416454 PMC7205693

[B107] Shu J. L. Zhang X. Z. Han L. Zhang F. Wu Y. J. Tang X. Y. (2019). Paeoniflorin-6'-O-benzene sulfonate alleviates collagen-induced arthritis in mice by downregulating BAFF-TRAF2-NF-κB signaling: comparison with biological agents. Acta Pharmacol. Sin. 40, 801–813. 10.1038/s41401-018-0169-5 30446734 PMC6786314

[B108] Skaug B. Jiang X. Chen Z. J. (2009). The role of ubiquitin in NF-kappaB regulatory pathways. Annu. Rev. Biochem. 78, 769–796. 10.1146/annurev.biochem.78.070907.102750 19489733

[B109] Sun S. C. (2011). Non-canonical NF-κB signaling pathway. Cell Res. 21, 71–85. 10.1038/cr.2010.177 21173796 PMC3193406

[B110] Sun S. C. (2017). The non-canonical NF-κB pathway in immunity and inflammation. Nat. Rev. Immunol. 17, 545–558. 10.1038/nri.2017.52 28580957 PMC5753586

[B111] Tang J. Cheng X. Yi S. Zhang Y. Tang Z. Zhong Y. (2021). Euphorbia factor L2 ameliorates the progression of K/BxN serum-induced arthritis by blocking TLR7 mediated IRAK4/IKKβ/IRF5 and NF-kB signaling pathways. Front. Pharmacol. 12, 773592. 10.3389/fphar.2021.773592 34950033 PMC8691750

[B112] Tanimura N. Saitoh S. Matsumoto F. Akashi-Takamura S. Miyake K. (2008). Roles for LPS-dependent interaction and relocation of TLR4 and TRAM in TRIF-signaling. Biochem. Biophys. Res. Commun. 368, 94–99. 10.1016/j.bbrc.2008.01.061 18222170

[B113] Tew X. N. Lau N. J. X. Chellappan D. K. Madheswaran T. Zeeshan F. Tambuwala M. M. (2020). Immunological axis of berberine in managing inflammation underlying chronic respiratory inflammatory diseases. Chemico-biological Interact. 317, 108947. 10.1016/j.cbi.2020.108947 31968208

[B114] Wagenlehner F. M. E. Dittmar F. (2022). Re: global burden of bacterial antimicrobial resistance in 2019: a systematic analysis. Eur. Urol. 82, 658. 10.1016/j.eururo.2022.08.023 36068104

[B115] Wang F. Han Y. Xi S. Lu Y. (2020). Catechins reduce inflammation in lipopolysaccharide-stimulated dental pulp cells by inhibiting activation of the NF-κB pathway. Oral Dis. 26 (0), 815–821. 10.1111/odi.13290 31999881

[B116] Wang J. Li F. Ding J. Tian G. Jiang M. Gao Z. (2016). Investigation of the anti-asthmatic activity of Oridonin on a mouse model of asthma. Mol. Med. Rep. 14, 2000–2006. 10.3892/mmr.2016.5485 27431862 PMC4991768

[B117] Wang Q. Zhou X. Yang L. Luo M. Han L. Lu Y. (2019). Gentiopicroside (GENT) protects against sepsis induced by lipopolysaccharide (LPS) through the NF-κB signaling pathway. Ann. Transl. Med. 7, 731. 10.21037/atm.2019.11.126 32042747 PMC6990009

[B118] Wang Q. Zhou X. Zhao Y. Xiao J. Lu Y. Shi Q. (2018a). Polyphyllin I ameliorates collagen-induced arthritis by suppressing the inflammation response in macrophages through the NF-κB pathway. Front. Immunol. 9, 2091. 10.3389/fimmu.2018.02091 30319603 PMC6170622

[B119] Wang S. Tian Y. Wang M. Wang M. Sun G. B. Sun X. B. (2018b). Advanced activity-based protein profiling application strategies for drug development. Front. Pharmacol. 9, 353. 10.3389/fphar.2018.00353 29686618 PMC5900428

[B120] Weiss J. Barker J. H. (2018). Diverse pro-inflammatory endotoxin recognition systems of mammalian innate immunity. F1000Research 7, 10.12688/f1000research.13977.1 PMC5931271 29770204

[B121] Wertz I. E. Dixit V. M. (2010). Signaling to NF-kappaB: regulation by ubiquitination. Cold Spring Harb. Perspect. Biol. 2, a003350. 10.1101/cshperspect.a003350 20300215 PMC2829959

[B122] Wooff J. Pastushok L. Hanna M. Fu Y. Xiao W. (2004). The TRAF6 RING finger domain mediates physical interaction with Ubc13. FEBS Lett. 566, 229–233. 10.1016/j.febslet.2004.04.038 15147900

[B123] Xia Z. P. Sun L. Chen X. Pineda G. Jiang X. Adhikari A. (2009). Direct activation of protein kinases by unanchored polyubiquitin chains. Nature 461, 114–119. 10.1038/nature08247 19675569 PMC2747300

[B124] Xu M. Skaug B. Zeng W. Chen Z. J. (2009). A ubiquitin replacement strategy in human cells reveals distinct mechanisms of IKK activation by TNFalpha and IL-1beta. Mol. Cell 36, 302–314. 10.1016/j.molcel.2009.10.002 19854138 PMC2779160

[B125] Yao X. Ding Z. Xia Y. Wei Z. Luo Y. Feleder C. (2012). Inhibition of monosodium urate crystal-induced inflammation by scopoletin and underlying mechanisms. Int. Immunopharmacol. 14, 454–462. 10.1016/j.intimp.2012.07.024 22914669

[B126] Ye H. Arron J. R. Lamothe B. Cirilli M. Kobayashi T. Shevde N. K. (2002). Distinct molecular mechanism for initiating TRAF6 signalling. Nature 418, 443–447. 10.1038/nature00888 12140561

[B127] Zanoni I. Ostuni R. Marek L. R. Barresi S. Barbalat R. Barton G. M. (2011). CD14 controls the LPS-induced endocytosis of Toll-like receptor 4. Cell 147, 868–880. 10.1016/j.cell.2011.09.051 22078883 PMC3217211

[B128] Zhang D. Li X. Hu Y. Jiang H. Wu Y. Ding Y. (2018). Tabersonine attenuates lipopolysaccharide-induced acute lung injury via suppressing TRAF6 ubiquitination. Biochem. Pharmacol. 154, 183–192. 10.1016/j.bcp.2018.05.004 29746822

[B129] Zhang H. Lang W. Wang S. Li B. Li G. Shi Q. (2020). Echinacea polysaccharide alleviates LPS-induced lung injury via inhibiting inflammation, apoptosis and activation of the TLR4/NF-κB signal pathway. Int. Immunopharmacol. 88, 106974. 10.1016/j.intimp.2020.106974 33182056

[B130] Zhang H. Shan Y. Wu Y. Xu C. Yu X. Zhao J. (2017). Berberine suppresses LPS-induced inflammation through modulating Sirt1/NF-κB signaling pathway in RAW264.7 cells. Int. Immunopharmacol. 52, 93–100. 10.1016/j.intimp.2017.08.032 28888780

[B131] Zhang K. Jiao X. F. Li J. X. Wang X. W. (2015a). Rhein inhibits lipopolysaccharide-induced intestinal injury during sepsis by blocking the toll-like receptor 4 nuclear factor-κB pathway. Mol. Med. Rep. 12, 4415–4421. 10.3892/mmr.2015.3925 26081522

[B132] Zhang L. Wang G. Hou W. Li P. Dulin A. Bonkovsky H. L. (2010). Contemporary clinical research of traditional Chinese medicines for chronic hepatitis B in China: an analytical review. Hepatology 51, 690–698. 10.1002/hep.23384 20101751 PMC2930399

[B133] Zhang N. Liu Z. Luo H. Wu W. Nie K. Cai L. (2019). FM0807 decelerates experimental arthritis progression by inhibiting inflammatory responses and joint destruction via modulating NF-κB and MAPK pathways. Biosci. Rep. 39. 10.1042/bsr20182263 PMC6722489 31431516

[B134] Zhang R. Ren S. Dai Q. Shen T. Li X. Li J. (2022). InflamNat: web-based database and predictor of anti-inflammatory natural products. J. Cheminform 14, 30. 10.1186/s13321-022-00608-5 35659771 PMC9167499

[B135] Zhang S. Xu P. Zhu Z. Zhou L. Li J. Zhou R. (2023). Acetylation of P65lys310 by P300 in macrophages mediates anti-inflammatory property of berberine. Redox Biol. 62, 102704. 10.1016/j.redox.2023.102704 37086629 PMC10172918

[B136] Zhang T. Wang J. Wang S. Ma C. (2015b). Timosaponin B-II inhibits lipopolysaccharide-induced acute lung toxicity via TLR/NF-κB pathway. Toxicol. Mech. Methods 25, 665–671. 10.3109/15376516.2015.1045652 26540118

[B137] Zhao B. Zhang Q. Liang X. Xie J. Sun Q. (2021). Quercetin reduces inflammation in a rat model of diabetic peripheral neuropathy by regulating the TLR4/MyD88/NF-κB signalling pathway. Eur. J. Pharmacol. 912, 174607. 10.1016/j.ejphar.2021.174607 34743981

[B138] Zhao H. Zheng Q. Hu X. Shen H. Li F. (2016). Betulin attenuates kidney injury in septic rats through inhibiting TLR4/NF-κB signaling pathway. Life Sci. 144, 185–193. 10.1016/j.lfs.2015.12.003 26656467

[B139] Zhong J. Wang F. Wang Z. Shen C. Zheng Y. Ma F. (2019). Aloin attenuates cognitive impairment and inflammation induced by d-galactose via down-regulating ERK, p38 and NF-κB signaling pathway. Int. Immunopharmacol. 72, 48–54. 10.1016/j.intimp.2019.03.050 30959371

[B140] Zhou J. Deng Y. Li F. Yin C. Shi J. Gong Q. (2019). Icariside II attenuates lipopolysaccharide-induced neuroinflammation through inhibiting TLR4/MyD88/NF-κB pathway in rats. Biomed. Pharmacother. 111, 315–324. 10.1016/j.biopha.2018.10.201 30590319

[B141] Zhu Y. Ouyang Z. Du H. Wang M. Wang J. Sun H. (2022). New opportunities and challenges of natural products research: when target identification meets single-cell multiomics. Acta Pharm. Sin. B 12, 4011–4039. 10.1016/j.apsb.2022.08.022 36386472 PMC9643300

[B142] Zou Y. F. Li C. Y. Fu Y. P. Jize X. P. Zhao Y. Z. Peng X. (2023). Angelica sinensis aboveground part polysaccharide and its metabolite 5-MT ameliorate colitis via modulating gut microbiota and TLR4/MyD88/NF-κB pathway. Int. J. Biol. Macromol. 242, 124689. 10.1016/j.ijbiomac.2023.124689 37148926

